# Polysulfide-1-oxides react with peroxyl radicals as quickly as hindered phenolic antioxidants and do so by a surprising concerted homolytic substitution†
†Electronic supplementary information (ESI) available: NMR spectra of new compounds, KIE experiments, details of LFP experiments and optimized geometries and energies for computational results. See DOI: 10.1039/c6sc01434h



**DOI:** 10.1039/c6sc01434h

**Published:** 2016-06-23

**Authors:** Jean-Philippe R. Chauvin, Evan A. Haidasz, Markus Griesser, Derek A. Pratt

**Affiliations:** a Department of Chemistry and Biomolecular Sciences , University of Ottawa , 10 Marie Curie Pvt. , Ottawa , Ontario , Canada . Email: dpratt@uottawa.ca ; Fax: +1-613-562-5170 ; Tel: +1-613-562-5800 ext. 2119

## Abstract

Polysulfides are important additives to a wide variety of industrial and consumer products and figure prominently in the chemistry and biology of garlic and related medicinal plants. Although their antioxidant activity in biological contexts has received only recent attention, they have long been ascribed ‘secondary antioxidant’ activity in the chemical industry, where they are believed to react with the hydroperoxide products of autoxidation to slow the auto-initiation of new autoxidative chain reactions. Herein we demonstrate that the initial products of trisulfide oxidation, trisulfide-1-oxides, are surprisingly reactive ‘primary antioxidants’, which slow autoxidation by trapping chain-carrying peroxyl radicals. In fact, they do so with rate constants (*k* = 1–2 × 10^4^ M^–1^ s^–1^ at 37 °C) that are indistinguishable from those of the most common primary antioxidants, *i.e.* hindered phenols, such as BHT. Experimental and computational studies demonstrate that the reaction occurs by a concerted bimolecular homolytic substitution (S_H_^2^), liberating a perthiyl radical – which is *ca.* 16 kcal mol^–1^ more stable than a peroxyl radical. Interestingly, the (electrophilic) peroxyl radical nominally reacts as a nucleophile – attacking the  of the trisulfide-1-oxide – a role hitherto suspected only for its reactions at metal atoms. The analogous reactions of trisulfides are readily reversible and not kinetically competent to inhibit hydrocarbon autoxidation, consistent with the longstanding view that organosulfur compounds must be oxidized to afford significant antioxidant activity. The reactivity of trisulfides and their oxides are contrasted with what is known of their shorter cousins and predictions are made and tested with regards to the reactivity of higher polysulfides and their 1-oxides – the insights from which may be exploited in the design of future antioxidants.

## Abstract 2

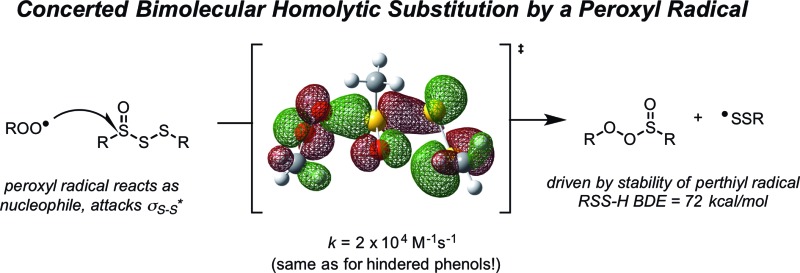
Polysulfides, important industrial additives and curious natural products, are activated toward substitution by peroxyl radicals upon oxidation to polysulfide-1-oxides.

## Introduction

**Scheme 1:**
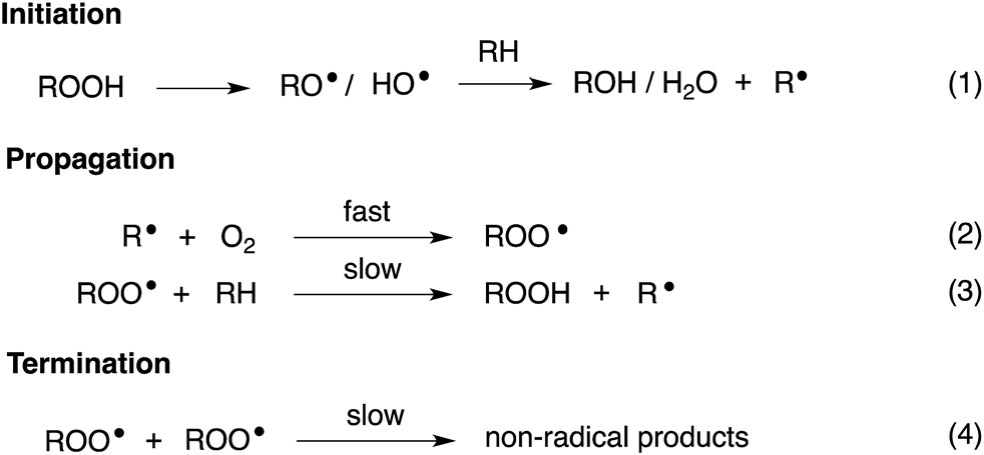
Hydrocarbon autoxidation.

Organosulfur compounds have long been recognized to slow hydrocarbon autoxidation, the deleterious free radical chain reaction depicted in Scheme 1.^1^ In fact, it was the pioneering work of Denison and Condit^2^ in the 1940s that lead to the conclusion that “*the oxidation stability of refined petroleum lubricating oils is the result of small quantities of natural sulfur compounds and not of any inherent stability of the hydrocarbon fraction itself. In the absence of the natural sulfur compounds the hydrocarbon fraction oxidizes rapidly and, in the initial stages of the reaction, autocatalytically*”. Hence, although naturally-occurring organosulfur compounds are removed from crude oil in its refinement, organosulfur compounds comprise important additives to finished products.^3^

**Scheme 2:**
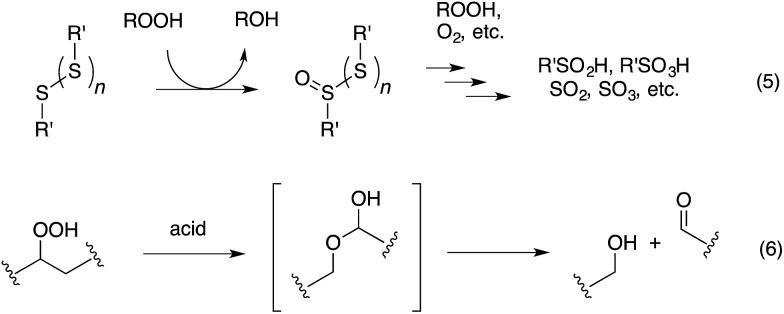
Key reactions of organosulfur antioxidants.

The antioxidant mechanisms of organosulfur compounds are often presented as complex. However, it is generally acknowledged that they must first be oxidized to render them effective.^4^ Organosulfur compounds are ascribed ‘secondary antioxidant’ activity since it is believed that they slow the rate of initiation (eqn (1)) by decomposing hydroperoxides.^5^ Two mechanisms are particularly important in this context: the direct reduction of hydroperoxides (eqn (5)) and the production of Brønsted or Lewis acids, which catalytically decompose hydroperoxides (eqn (6)) (Scheme 2).

Secondary antioxidants can be contrasted with ‘primary antioxidants’, which inhibit autoxidation directly *via* reaction with chain-propagating peroxyl radicals.^7,8^ The most common examples of primary antioxidants - generally referred to as radical-trapping antioxidants (RTAs) - are phenols and diarylamines.^6^

Organosulfur compounds may also act as primary antioxidants.^7,9^ Koelewijn and Berger^10^ showed that sulfides, upon oxidation to sulfoxides, undergo Cope-type elimination to yield a sulfenic acid (eqn (7)), which they surmised would undergo fast reactions with peroxyl radicals (eqn (8)). Since sulfenic acids are transient species that rapidly undergo self-reaction to yield thiosulfinates, they were unable to directly determine the kinetics of reaction (8), but estimated a rate constant of *ca.* 10^7^ M^–1^ s^–1^ based on inhibited autoxidations of tetralin with *in situ* formation of *t*-BuSOH from thermolysis of di-*tert*-butyl sulfoxide at 60 °C.^10^
7
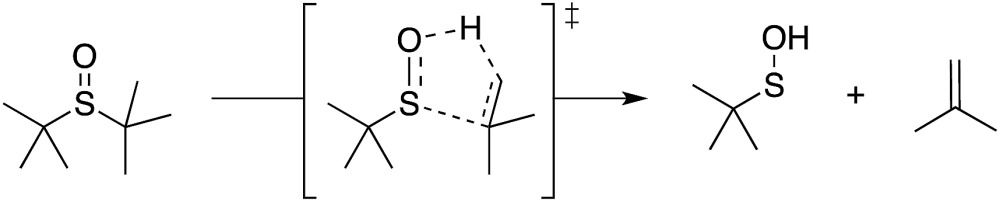

8
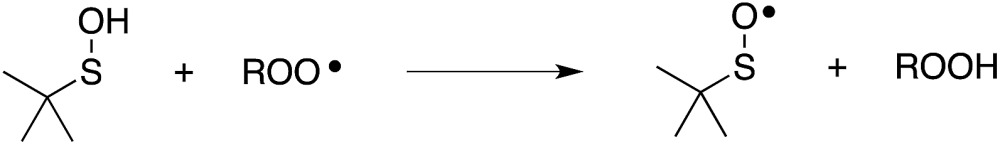



Using a persistent sulfenic acid, 9-triptycenesulfenic acid,^11^ we recently determined the kinetics and thermodynamics of the reaction of a sulfenic acid with peroxyl radicals directly. The weak O–H bond in the sulfenic acid (71.9 kcal mol^–1^)^12^ gives way to highly favourable reaction thermodynamics with peroxyl radicals (*ca.* 14 kcal mol^–1^), and the presence of the sulfur atom α to the reactive O–H drives a very fast proton-coupled electron transfer reaction with peroxyl radicals (*k* = 3 × 10^6^ M^–1^ s^–1^) – despite the steric demand of the triptycenyl moiety.^13,14^

Our studies of the radical chemistry of sulfenic acids were motivated by the widely reported antioxidant activity of allicin, the odorous thiosulfinate found in garlic, and petivericin, the analogous thiosulfinate from the medicinal plant *Petiveria alliacea*. We demonstrated^15,16^ that the antioxidant activity of both thiosulfinates was dependent on the Cope-type elimination of a sulfenic acid, which proceeds readily for both compounds at ambient temperature owing to the activated β C–H bond.^17^
9
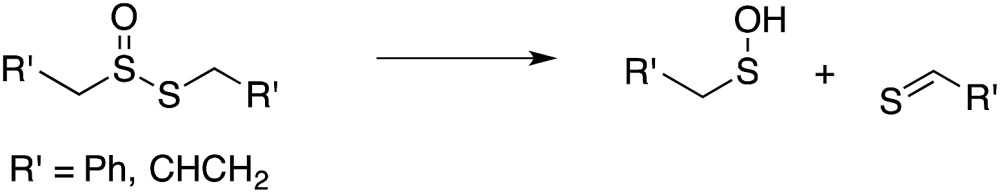

10
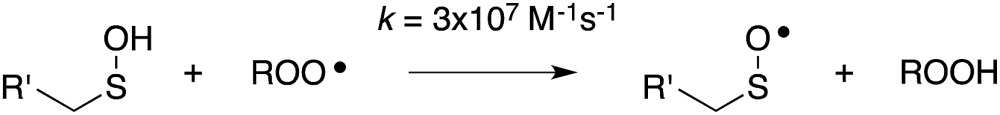



Since trisulfides and their oxides are among the most prevalent organosulfur compounds in *Allium* species (*e.g.*, diallyltrisulfide and diallyltrisulfide-1-oxide from garlic)^18,19^ and are predominant among the mixtures of sulfurized materials added to protect petroleum-derived products from autoxidation,^4,20^ we wondered if they would possess ‘primary antioxidant’ reactivity analogous to that of hindered sulfoxides and activated thiosulfinates. Herein we report an investigation of the RTA activities of some trisulfides and their 1-oxides in an attempt to extend the foregoing knowledge to a more comprehensive view of the primary antioxidant activity of polysulfides and their 1-oxides, in general.^21^

## Results

Given both the volatility and lability of diallyl trisulfide and its 1-oxide, their benzyl analogs, dibenzyl trisulfide (BnSSSBn) and dibenzyltrisulfide-1-oxide (BnS(O)SSBn), were investigated.^22^ These compounds, synthesized as described by Harpp^23^ and Stensaas,^24^ respectively, are solids at ambient temperature and allow direct comparison with petivericin (BnS(O)SBn) and its corresponding disulfide (BnSSBn). The reactivity of di-*tert*-butyltrisulfide (*t*-BuSSS*t*-Bu)^23^ and its 1-oxide (*t*-BuS(O)SS*t*-Bu)^25^ – also synthesized as described by Harpp – were also explored. These derivatives lack the activated β C–H in the allyl and benzyl moieties found in the garlic- and petiveria-derived allicin and petivericin, respectively.
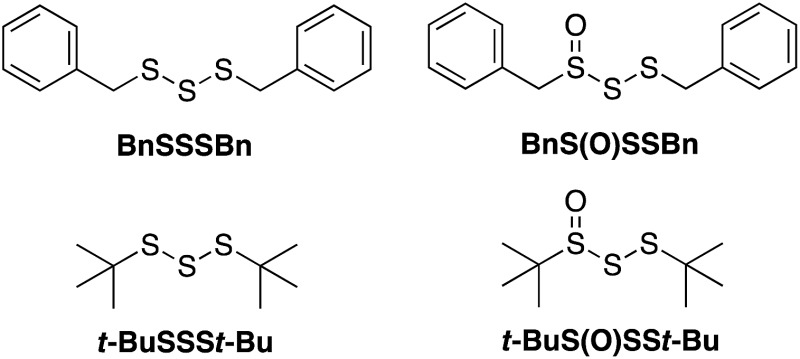


### I. Inhibited autoxidations

**Scheme 3:**
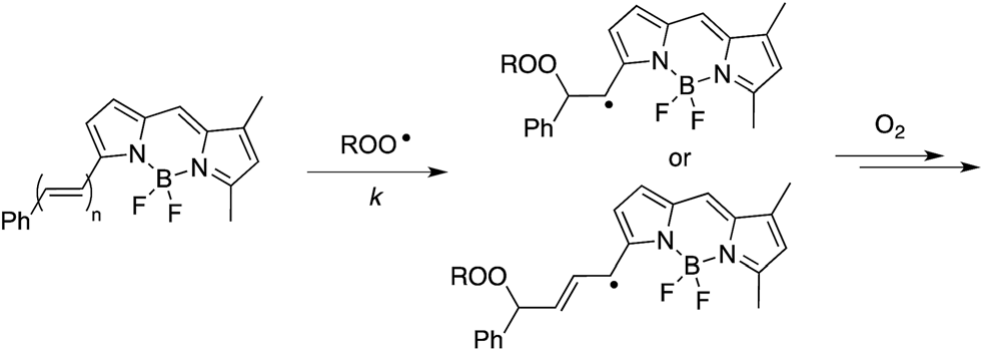
The absorbance of STY-BODIPY and PBD-BODIPY decrease as they are co-autoxidized with cumene and styrene, respectively. STY-BODIPY: *n* = 1, *λ*_max_ = 591 nm, *k* = 141 M^–1^ s^–1^; PBD-BODIPY: *n* = 2, *λ*_max_ = 571 nm, *k* = 2720 M^–1^ s^–1^.

**Fig. 1:**
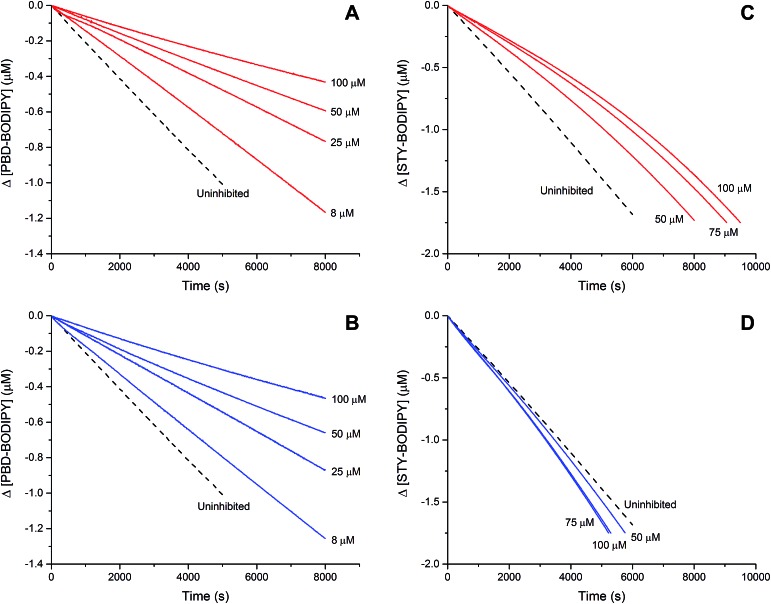
Thermally-initiated (AIBN, 6 mM) co-autoxidation of styrene (4.4 M) and PBD-BODIPY (10 μM) at 37 °C in chlorobenzene in the presence of either BnS(O)SSBn (A) or *t*-BuS(O)SS*t*-Bu (B). Corresponding data is shown alongside for the AIBN-initiated (40 mM) co-autoxidation of cumene (3.6 M) and STY-BODIPY (10 μM) in the presence of BnS(O)SSBn (C) or *t*-BuS(O)SS*t*-Bu (D).

The efficacies of the trisulfide-1-oxides as inhibitors of hydrocarbon autoxidation were investigated using styrene and cumene as model substrates. Although autoxidations are conventionally monitored by O_2_ consumption, the recently developed method based on the competitive autoxidation of either PBD-BODIPY (with styrene) or STY-BODIPY (with cumene) was instead employed (Scheme 3).^26^ Representative results are shown in Fig. 1A and B for varying amounts of BnS(O)SSBn and *t*-BuS(O)SS*t*-Bu.

The data in Fig. 1A and B clearly demonstrate the ability of both trisulfide-1-oxides to retard the oxidation – and to a similar extent. From the rates of the retarded reactions, and assuming a stoichiometric factor of *n* = 1 (*vide infra*), the inhibition rate constants for BnS(O)SSBn and *t*-BuS(O)SS*t*-Bu were calculated using eqn (11) to be *k*_inh_ = (1.5 ± 0.4) × 10^4^ M^–1^ s^–1^ and (1.1 ± 0.3) × 10^4^ M^–1^ s^–1^, respectively. The corresponding disulfides and trisulfides had no effect on the rate of PBD-BODIPY oxidation.11
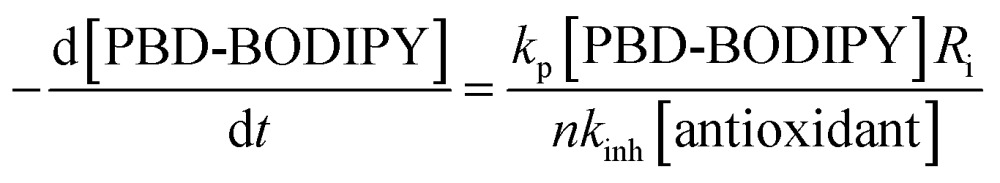



The lack of clear inhibited periods for the trisulfide-1-oxides in the foregoing styrene autoxidations precluded a determination of the stoichiometry for their reaction with peroxyl radicals. Therefore, co-autoxidations of cumene and STY-BODIPY were also carried out. These less oxidizable substrates generally provide greater resolution between inhibited and uninhibited phases of the autoxidation.^26^ The results are shown for *t*-BuS(O)SS*t*-Bu and BnS(O)SSBn in Fig. 1C and D, respectively.

The STY-BODIPY/cumene co-autoxidations were less well inhibited by the trisulfide-1-oxides compared to the PBD-BODIPY/styrene co-autoxidations; in fact, *t*-BuS(O)SS*t*-Bu was unable to inhibit the autoxidation at all, displaying instead a slight, but reproducible, acceleration of the autoxidation.^27^ This was particularly surprising given the greater propagation rate constants of PBD-BODIPY (2720 M^–1^ s^–1^) and styrene (41 M^–1^ s^–1^) compared to STY-BODIPY (141 M^–1^ s^–1^) and cumene (0.34 M^–1^ s^–1^). Kinetic analysis of the initial rates of the BnS(O)SSBn-inhibited autoxidations (*i.e.*eqn (11)) afforded *k*_inh_ = (2.4 ± 0.6) × 10^3^ M^–1^ s^–1^; *ca.* 6-fold smaller than the rate constant derived from the styrene co-autoxidations. Nevertheless, the presence of a distinct inhibited period followed by reaction progress at the same rate as in the uninhibited reaction enabled the determination of the stoichiometry from the length of the inhibited period, *τ* (as in eqn (12)). The result was *n* = 0.9 ± 0.2, thereby validating the assumption made in the styrene autoxidations (*vide supra*).12
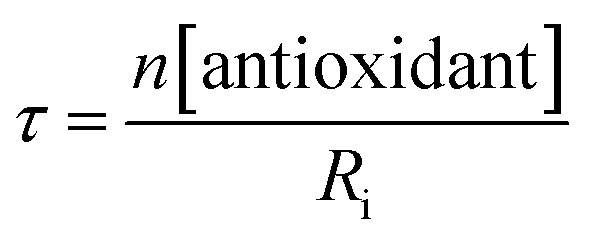



To probe whether the benzylic H-atoms in BnS(O)SSBn play a role in its radical-trapping antioxidant activity, *d*_4_-BnS(O)SSBn was synthesized in a manner similar to that used to prepare *d*_4_-BnS(O)SBn in our previous work (see Experimental section),^13^ and its RTA activity was evaluated in styrene/PBD-BODIPY co-autoxidations. Experiments were also carried out wherein a small volume of a protic/deuteric solvent was added to determine if an exchangeable H-atom/D-atom is involved in the radical-trapping reaction. The results are given in Table 1 (see ESI† for the inhibited autoxidation traces). For comparison, *k*_H_/*k*_D_ values of 16 and 18 were determined from *d*_4_-BnS(O)SBn-inhibited autoxidations carried out in the presence of D_2_O.^13^ 
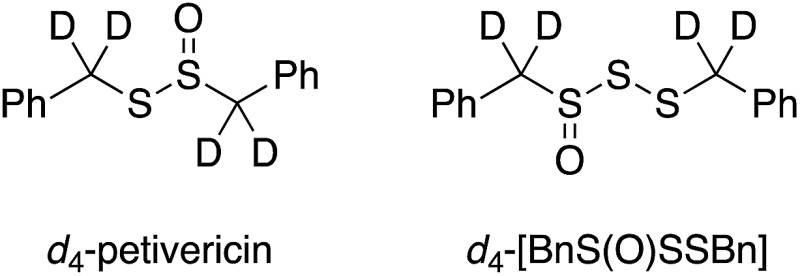


**Table 1:** Inhibition rate constants determined from thermally-initiated co-autoxidation of styrene and PBD-BODIPY in chlorobenzene at 37 °C in the presence of either BnS(O)SSBn or *d*_4_-BnS(O)SSBn and MeOH or MeOD

Trisulfide-1-oxide/additive	*k*_inh_ (M^–1^ s^–1^)	*k*_H_/*k*_D_
BnS(O)SSBn	(1.5 ± 0.4) × 10^4^	—
BnS(O)SSBn/MeOH (1%)	(1.5 ± 0.4) × 10^4^	—
BnS(O)SSBn/MeOD (1%)	(1.3 ± 0.4) × 10^4^	1.2
*d*_4_-BnS(O)SSBn/MeOD (1%)	(1.1 ± 0.2) × 10^4^	1.4

Analogous co-autoxidations inhibited by *t*-BuS(O)SS*t*-Bu in the presence of either 1% MeOH or MeOD yielded indistinguishable results from co-autoxidations carried out in their absence.

### II. Computational studies

**Scheme 4:**
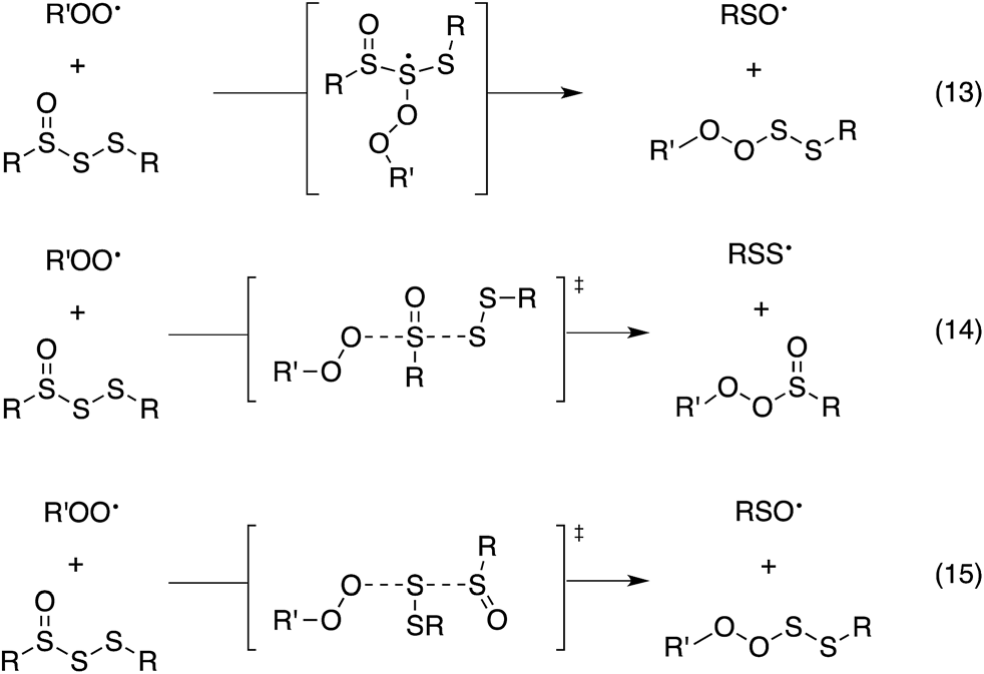
Mechanistic possibilities for the reaction of trisulfide-1-oxides with peroxyl radicals.

Since the similar reactivity of BnS(O)SSBn and *t*-BuS(O)SS*t*-Bu toward styrene-derived peroxyl radicals suggested no role for the substituents on the trisulfide-1-oxide, computational studies focused on the direct interaction of peroxyl radicals and the trisulfide-1-oxide moiety. Three possible mechanisms were considered, which are shown in Scheme 4. The first was a step-wise addition/fragmentation mechanism, which liberates a sulfinyl radical along with formation of a peroxydisulfane (eqn (13)). The second and third mechanisms are concerted homolytic substitutions occurring on either the sulfinyl sulfur (S1), to liberate a perthiyl radical (eqn (14)), or on the adjacent sulfur atom (S2), to liberate a sulfinyl radical (eqn (15), the concerted analog of eqn (13)).

**Fig. 2:**
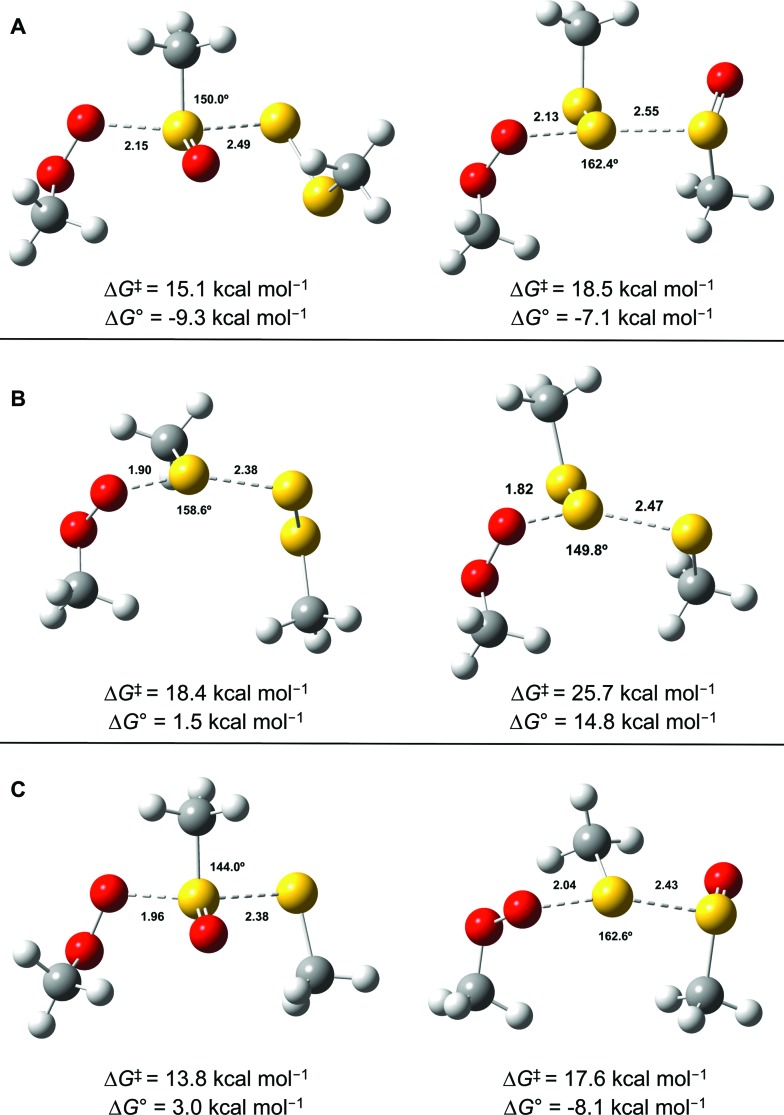
Transition state structures for the reaction of MeOO˙ with MeS(O)SSMe at either the sulfinyl sulfur (S1, left) or S2 (A), MeSSSMe at either S1 (left) or S2 (B) and MeS(O)SMe at either the sulfinyl sulfur (S1, left) or S2 (C). Bond lengths (in Å) are shown for the forming O–S and breaking S–S bonds. The angle between these bonds is also indicated.

CBS-QB3 calculations^28^ were carried out on model compounds wherein the substituents on the trisulfide-1-oxide and peroxyl radical (R and R′, respectively) were methyl groups. Despite extensive effort, a minimum energy structure for the intermediate in the step-wise reaction (eqn (13)) could not be located. However, transition states for both concerted homolytic substitutions were readily located, and are shown in Fig. 2A. The structures are 15.1 and 18.5 kcal mol^–1^, respectively, higher in free energy than the separated reactants. Application of transition state theory affords rate constants of 3.8 × 10^3^ and 14 M^–1^ s^–1^, respectively – the former in good agreement with the experimental data for BnS(O)SSBn of (1.5 ± 0.4) × 10^4^. Replacement of the methyl substituents on the trisulfide-1-oxide and methylperoxyl radical with *t*-butyl groups increases the barrier by 3.7 kcal mol^–1^ – consistent with the fact that *t*-BuS(O)SS*t*-Bu does not inhibit cumene autoxidation.^29^

**Fig. 3:**
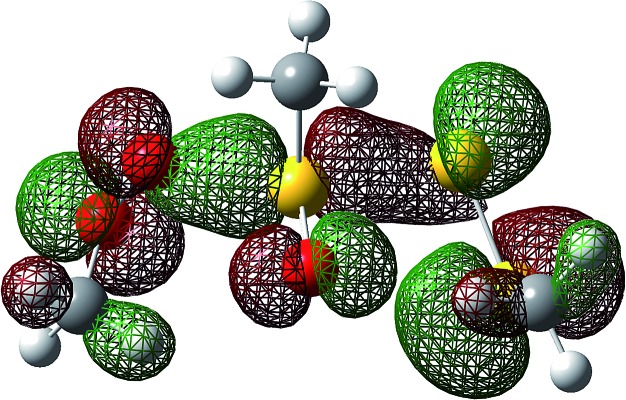
The highest occupied molecular orbital for the lowest energy transition state structure for the reaction of MeOO˙ and MeS(O)SSMe illustrates the key interactions between the π* orbitals of the peroxyl (left), sulfinyl (centre) and perthiyl (right) moieties.

Both substitutions are predicted to be thermodynamically favourable: Δ*G* ^0^ = –9.3 kcal mol^–1^ for reaction at S1 (the sulfinyl sulfur) and Δ*G* ^0^ = –7.1 kcal mol^–1^ for reaction at S2. Much of the energetic benefit derives from exchange of the peroxyl radical for either of the more stabilized perthiyl or sulfinyl radicals – which is clear when one considers the X–H BDEs (calculated by CBS-QB3) in MeSS–H (70.8 kcal mol^–1^) and MeSO–H (68.4 kcal mol^–1^) compared to MeOO–H (86.2 kcal mol^–1^) – as well as the stronger S–O bonds in the products (40.1 and 37.2 kcal mol^–1^ in MeOOS(O)Me and MeOOSSMe compared to 30.6 kcal mol^–1^ for the S1–S2 bond in MeS(O)SSMe). In the reaction at S1, the sulfinyl S–O bond length (1.480 Å) contracts ever so slightly from the starting material (1.487 Å), whereas in the reaction at S2, the sulfinyl S–O lengthens slightly (1.496 Å) on its way to becoming a free sulfinyl radical (1.511 Å). Consideration of the transition state structure suggests that the lone pair on the S1 sulfur does not participate directly in the reaction. In fact, the electronic structure of the transition state can essentially be viewed as interacting peroxyl, sulfinyl and perthiyl radicals (Fig. 3).

This view is reinforced by the results of natural bond orbital (NBO) analyses^30^ of the corresponding wavefunction, which is dominated by peroxyl π* (SOMO) → 
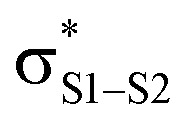
 and perthiyl π* (SOMO) → 
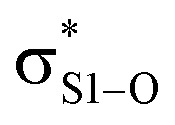
 interactions – worth a total of 73.5 kcal mol^–1^. Interestingly, the NBO analyses also reveal strong interactions between the lone pairs on the sulfinyl oxygen and the 
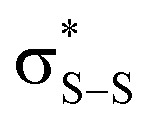
 and 
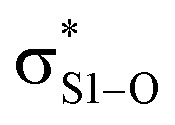
 orbitals – worth a total of 60.2 kcal mol^–1^ – consistent with the fact that the transition state is stabilized by oxidation of S1.

For comparison, the analogous transition states for the reaction of a trisulfide with a peroxyl radical were determined and are shown in Fig. 2B. These structures are 18.4 and 25.7 kcal mol^–1^, respectively, higher in energy than the separated reactants. The corresponding rate constants determined by transition state theory are 17 and 1.2 × 10^–4^ M^–1^ s^–1^, respectively – consistent with the fact that no inhibition of styrene or cumene autoxidation was observed in the presence of the trisulfides. Both of these reactions are endergonic, with reaction at S1 being far less unfavourable (Δ*G* ^0^ = +1.5 kcal mol^–1^), than that at S2 (Δ*G* ^0^ = +14.8 kcal mol^–1^). This is largely due to the greater stability of the perthiyl radical leaving group in the former reaction relative to the thiyl radical leaving group in the latter reaction (the CBS-QB3-calculated MeS–H and MeSS–H BDEs are 87.1 and 70.8 kcal mol^–1^, respectively).

Although analogous transition state structures could not be located for the reaction of a disulfide with a peroxyl radical, transition state structures were again readily located for the reaction of a peroxyl radical with a thiosulfinate, and are shown in Fig. 2C. These structures are 13.8 and 17.6 kcal mol^–1^, respectively, higher in energy than the separated reactants. Interestingly, the barrier for reaction at S1 is even lower than that determined for the reaction of peroxyl radicals with the trisulfide-1-oxide. However, this reaction is endergonic by 3.0 kcal mol^–1^ – implying that it is readily reversible – while the reaction of the trisulfide-1-oxide is exergonic by 9.3 kcal mol^–1^. This difference arises largely since the reaction of the thiosulfinate produces a thiyl radical, which is far less stable than the perthiyl radical (by 16.3 kcal mol^–1^, *vide supra*). Although the reaction of the thiosulfinate at S2 is significantly exergonic (Δ*G* ^0^ = –8.1 kcal mol^–1^), it has a higher barrier. The rate constant calculated for substitution at S2 is 61 M^–1^ s^–1^, in reasonable agreement with rate constants determined experimentally for unactivated thiosulfinates (*e.g. n*-propyl *n*-propanethiosulfinate, for which *k*_inh_ = (244 ± 31) M^–1^ s^–1^).^31^

### III. Peroxyl radical-trapping by hydropersulfides

**Fig. 4:**
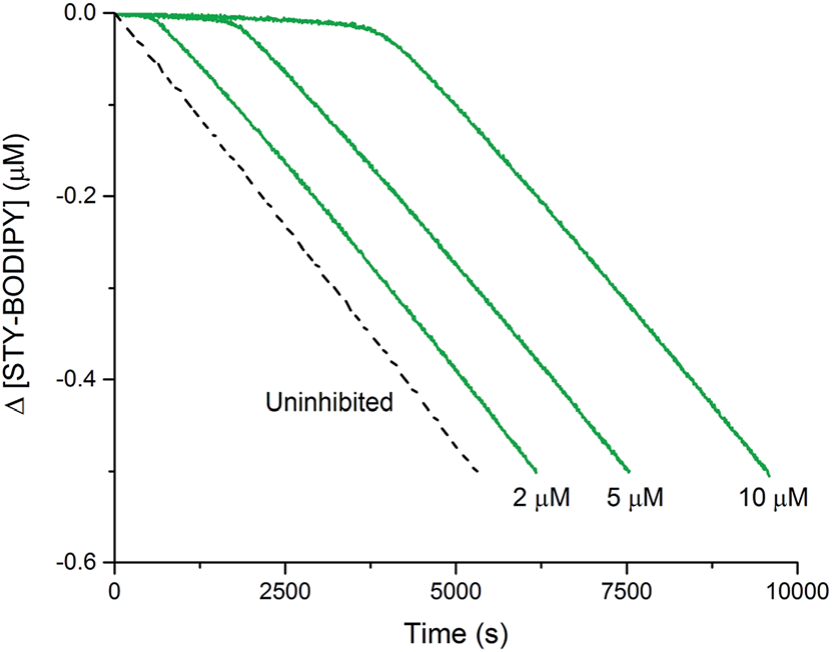
Thermally-initiated (AIBN, 6 mM) co-autoxidation of cumene (3.6 M) and STY-BODIPY (10 μM) at 37 °C in chlorobenzene in the presence of various concentrations of BnSSH.

The foregoing computational results imply that the preferred reaction path between trisulfide-1-oxide and peroxyl radical yields a perthiyl radical. The stoichiometry observed in the inhibited autoxidations (*n* = 1, *vide supra*) indicates that the perthiyl radical is unreactive under the autoxidation conditions. To confirm this point, benzyl hydropersulfide (BnSSH) was synthesized^32^ and its radical-trapping antioxidant activity investigated using the same STY-BODIPY/cumene co-autoxidation as for BnS(O)SSBn (*cf.* Fig. 1C). The results are shown in Fig. 4.

The stoichiometry of the reaction of BnSSH with peroxyl radicals was determined to be *n* = 1.0 ± 0.2 from this data, consistent with a mechanism involving initial H-atom transfer from the hydropersulfide to a chain-carrying peroxyl radical and then no involvement of the resultant perthiyl radical in the reaction. It should be noted that hydropersulfides are evidently very potent radical-trapping antioxidants – similar in reactivity to α-tocopherol, Nature's premier lipid-soluble radical-trapping antioxidant^33,34^ – a result we will follow up on in subsequent work.

### IV. Direct measurements of the fate of perthiyl radicals

The reaction of perthiyl radicals with O_2_ has been reported in aqueous solution to occur with *k* = 5.1 × 10^6^ M^–1^ s^–1^.^35^ We initially expected that the resulting RSSOO˙ may participate in the ensuing radical chemistry in the autoxidations inhibited by the trisulfide-1-oxide (and hydropersulfide). However, the foregoing results imply that RSSOO˙, if formed, does not participate. It may also be that this radical is either not formed under the reaction conditions or that the reaction is readily reversible.

To provide insight on these possibilities, the reactivity of perthiyl radicals towards oxygen was investigated in chlorobenzene by laser flash photolysis. Di-*tert*-butyl tetrasulfide was used as a model compound, since it is known to yield a pair of perthiyl radicals upon UV irradiation.^36^
16
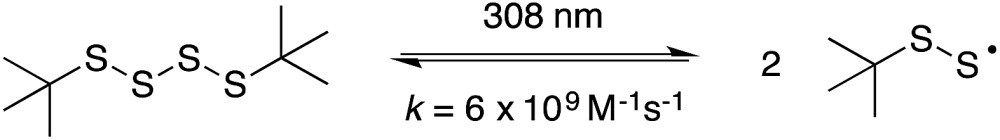



**Fig. 5:**
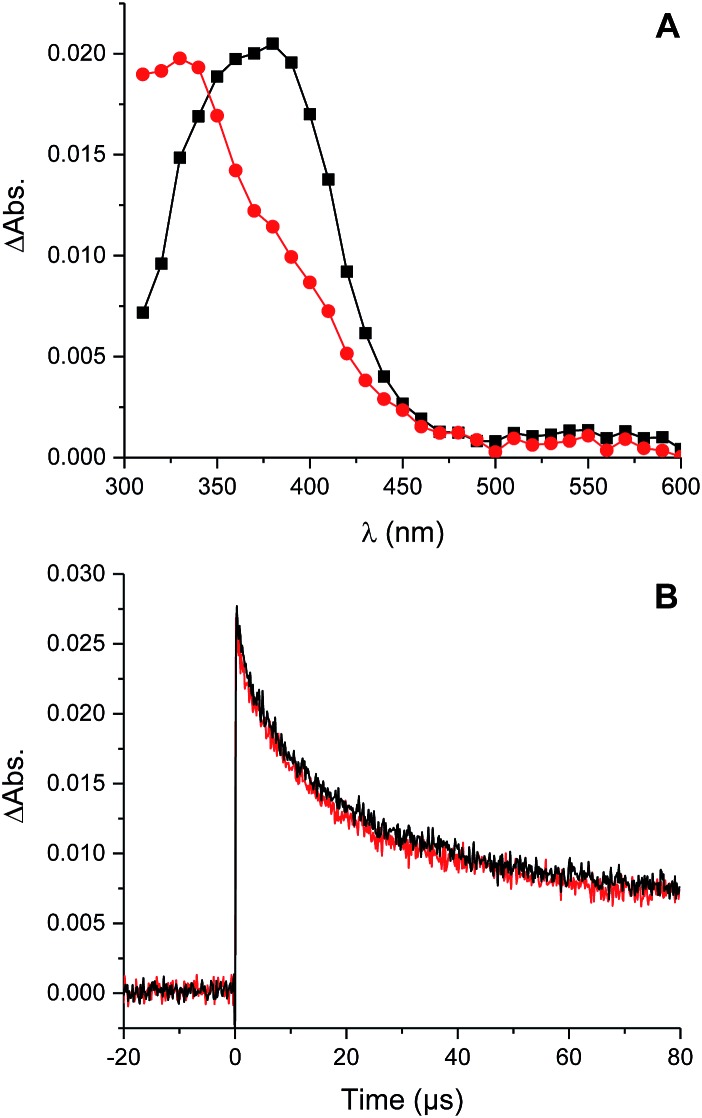
Transient absorption spectra obtained either 1 μs (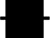) or 30 μs (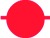) after photolysis of a 0.2 mM solution of di-*tert*-butyl tetrasulfide in chlorobenzene (A) and decay of the absorption at 390 nm in the presence (black) and absence (red) of O_2_ (B).

Photolysis of the tetrasulfide at 308 nm yielded a transient species with a pronounced absorption at 375 nm corresponding to the perthiyl radical (Fig. 5A). This transient absorption decayed rapidly (Fig. 5B) with concomitant reformation of the tetrasulfide at 340 nm (Fig. 5A).

The second order rate constant for the recombination of perthiyl radicals to the tetrasulfide was determined to be (6.0 ± 0.5) × 10^9^ M^–1^ s^–1^ at 25 °C (assuming *ε*_390 nm_ = 1600 M^–1^ cm^–1^ based on pulse radiolysis studies of similar compounds^35,37,38^). This is *ca.* 4-fold larger than the reported values in water (1.5 and 1.9 × 10^9^ M^–1^ s^–1^, respectively). Importantly, as is clear in Fig. 5B, no difference in the decay kinetics of the perthiyl radical could be detected in the presence or absence of O_2_.

We also carried out a series of computations to probe the plausibility of the reaction of a perthiyl radical with O_2_ (eqn (17)). It was not possible to find a bound perthiyl-O_2_ adduct using the CBS-QB3 approach that we employed above (where geometries are obtained at the B3LYP/CBSB7 level of theory). However, when the calculations were carried out with basis sets that included diffuse functions on the heavy atoms, bound structures were located. The corresponding CBS-QB3(+)^39^ Δ*G* ^0^ was 12.9 kcal mol^–1^ with an associated barrier of Δ*G* ^‡^ = 14.2 kcal mol^–1^ – consistent with the lack of reactivity determined experimentally.17
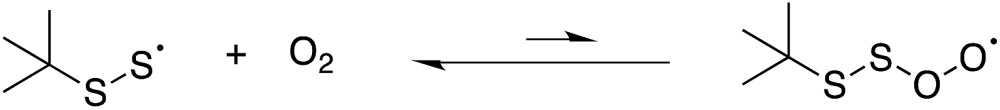



## Discussion

In line with the dogma that organosulfur compounds must be oxidized to afford antioxidant activity, trisulfides do not react at an appreciable rate with peroxyl radicals, while their 1-oxides are as reactive as hindered phenols, *e.g.*, *k*_inh_ = 1.5 × 10^4^ M^–1^ s^–1^ for BnS(O)SSBn and 1.4 × 10^4^ M^–1^ s^–1^ for 2,6-di-*tert*-butyl-4-methylphenol (BHT).^40^ Given the similar structure and reactivity of BnS(O)SSBn to petivericin, we initially wondered if it could undergo an analogous Cope-type elimination (eqn (18)) to afford a good H-atom donor. This would account for both its need to be oxygenated and its relatively high *k*_inh_ – which is too high for H-atom transfer from a benzylic C–H position (∼1 M^–1^ s^–1^).^41^
18
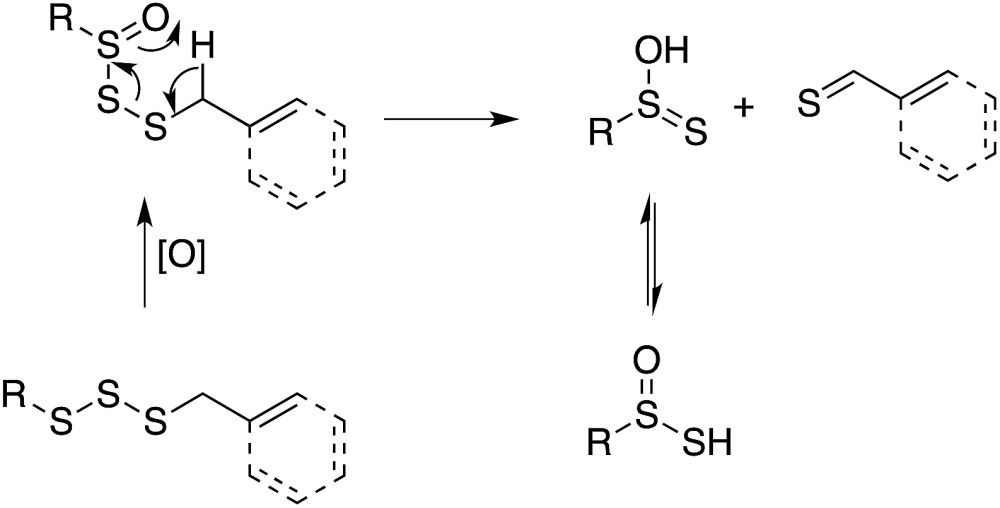



While a transition state for this process was readily located in CBS-QB3 calculations on the corresponding allylic trisulfide-1-oxide (the benzyl groups were exchanged for allyl groups for computational expediency), the structure was a prohibitive 40.9 kcal mol^–1^ higher in energy than the starting material. For comparison, the transition state structure for Cope-type elimination of 2-propenesulfenic acid from allicin is calculated to have Δ*H* ^‡^ = 19.5 kcal mol^–1^ (CBS-QB3), in good agreement with experimental observations.^15,16^

Of course, the fact that *t*-BuS(O)SS*t*-Bu reacts with styrylperoxyl radicals with essentially the same rate constant as BnS(O)SSBn implies that the mechanism involves direct reaction at the trisulfide-1-oxide moiety. We initially surmised that this could take place by an addition/fragmentation mechanism, where the peroxyl radical would add to a lone pair on the sulfur atom to form an intermediate sulfur-centered radical that would be capto-datively stabilized by the sulfur atom on one side and the sulfinyl group on the other (eqn (13)). Subsequent α-cleavage would yield either of two potential pairs of formal substitution products. However, computational efforts to provide evidence for this intermediate were unsuccessful.

Since computations indicated that the S(O)–SS bonds in trisulfide-1-oxides are weak (*i.e.* 29.8 kcal mol^–1^ in *t*-BuS(O)SS*t*-Bu by CBS-QB3 – see ESI†), a concerted substitution was considered next. This seemed unlikely since it requires that the peroxyl radical serve nominally as the nucleophile. Nevertheless, transition state structures were readily located for substitution at each of the sulfinyl sulfur atom (S1) and S2. Substitution at S1 was predicted to take place with a lower barrier (15.1 kcal mol^–1^ *vs.* 18.5 kcal mol^–1^ for substitution at S2) and the corresponding rate constant obtained from application of transition state theory was in good agreement with our experiments. This mechanism is also consistent with the lack of deuterium kinetic isotope effects on peroxyl radical-trapping by either *t*-BuS(O)SS*t*-Bu (*k*_H_/*k*_D_ = 1.0) or BnS(O)SSBn (*k*_H_/*k*_D_ = 1.4). This is in sharp contrast with the large KIEs observed for petivericin (*k*_H_/*k*_D_ = 16 or 18.2, depending on experimental approach) – whose mechanism involves two isotope-sensitive steps: C–H bond cleavage in the Cope-type elimination and O–H bond cleavage in the formal H-atom transfer from the sulfenic acid to the peroxyl radical.^13^

The rates of reactions of peroxyl radicals with RTAs are generally independent of the structure of the peroxyl radical; that is, inhibited autoxidations of styrene or cumene, where the chain reaction is carried by styrylperoxyl or cumylperoxyl radicals, respectively, usually yield the same inhibition rate constant.^40^ The fact that the trisulfide-1-oxides react at different rates with these peroxyl radicals, and that the steric demand of the substituents on the trisulfide-1-oxide have a greater impact on the rate when the peroxyl radical is also hindered (cumylperoxyl), further support that these reactions take place by a homolytic substitution mechanism at the sulfinyl sulfur atom.

**Scheme 5:**
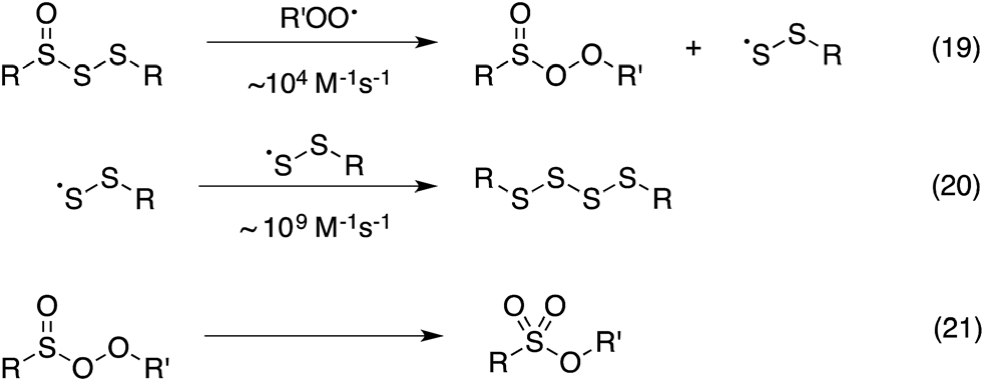
Proposed reaction mechanism for the trapping of peroxyl radicals by trisulfide-1-oxides.

Thus, the RTA activity of the trisulfide-1-oxides in organic solution can be ascribed to the reactions in Scheme 5. The substitution products are a perthiyl radical and a peroxysulfinate ester (eqn (19)). Interestingly, in contrast with the literature precedent (in aqueous solution), our flash photolysis experiments indicate that the perthiyl radical rapidly combines with another of itself to form a tetrasulfide (eqn (20)) even in the presence of O_2_. The peroxysulfinate ester is the same product that is formed upon reaction of a sulfinyl radical with a peroxyl radical, and is believed to rapidly rearrange to a sulfonate ester (eqn (21)) – possibly by O–O cleavage and in-cage recombination.^13,16^ This reaction mechanism is consistent with all of the foregoing data, including the fact that a trisulfide-1-oxide traps a single peroxyl radical under ambient conditions, unlike activated thiosulfinates and sulfoxides, which trap 2.

The reactions of peroxyl radicals at metal centers (*e.g.* magnesium, zinc, aluminum and tin) have been proposed to occur *via* an S_H_^2^ mechanism,^42^ but to the best of our knowledge, this mechanism does not seem to be operative on non-metal centers. Substitution of an alkyl substituent for a peroxyl substituent is commonly invoked in the autoxidation of trialkylboranes (*e.g.* in the common Et_3_B/O_2_ radical initiator system),^43^ but this is likely to be mechanistically distinct owing to the initial formation of a Lewis acid–base complex between a lone-pair on the peroxyl radical and the vacant boron 2p orbital that precedes homolytic boron–carbon bond cleavage.^44^ The substitutions of peroxyl radicals at centres that have non-bonded electron pairs, such as in phosphines and sulfides, are believed to proceed *via* addition–fragmentation sequences in non-polar solvents^45^ and by electron transfer in polar solvents when the peroxyl is highly electrophilic (*i.e.* halogenated methylperoxyls),^46,47^ respectively.

**Fig. 6:**
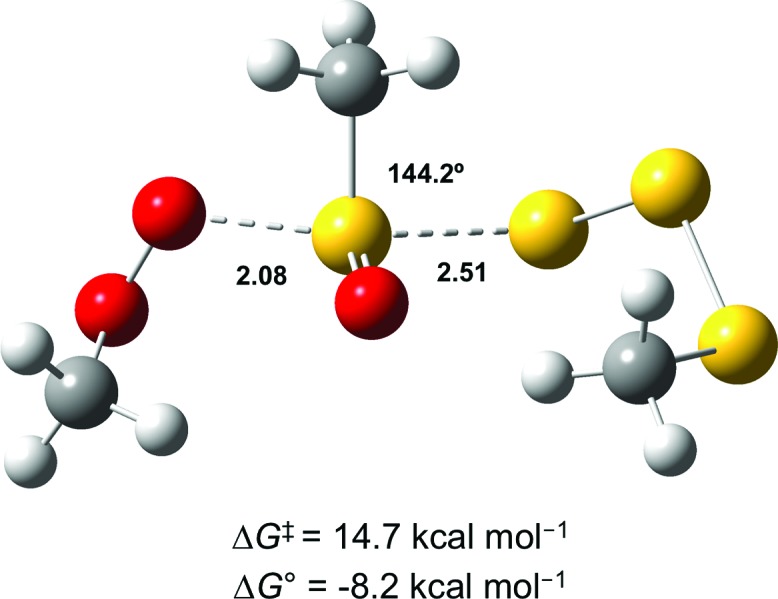
Transition state structure for the reaction of MeOO˙ with MeS(O)SSSMe. Bond lengths (in Å) are shown for the forming O–S and breaking S–S bonds. The angle between these bonds is also indicated.

In light of the mechanism in Scheme 5, the foregoing investigations were extended to include the next polysulfide-1-oxide. Initial CBS-QB3 calculations predicted Δ*G* ^0^ = –8.2 kcal mol^–1^ for MeOO˙ + MeS(O)SSSMe – little different from the –9.3 kcal mol^–1^ calculated for MeOO˙ + MeS(O)SSMe. This is explained simply on the basis that the leaving groups (MeSSS˙ and MeSS˙) are similarly stabilized (CBS-QB3 BDEs in MeSS–H and MeSSS–H are 70.8 and 72.7 kcal mol^–1^, respectively). Moreover, the transition state for the S_H_^2^ reaction of the tetrasulfide-1-oxide was readily located (*cf.* Fig. 6), and its associated Δ*G* ^‡^ = 14.7 kcal mol^–1^ was essentially the same as that which was calculated for the corresponding trisulfide-1-oxide (15.1 kcal mol^–1^).

**Fig. 7:**
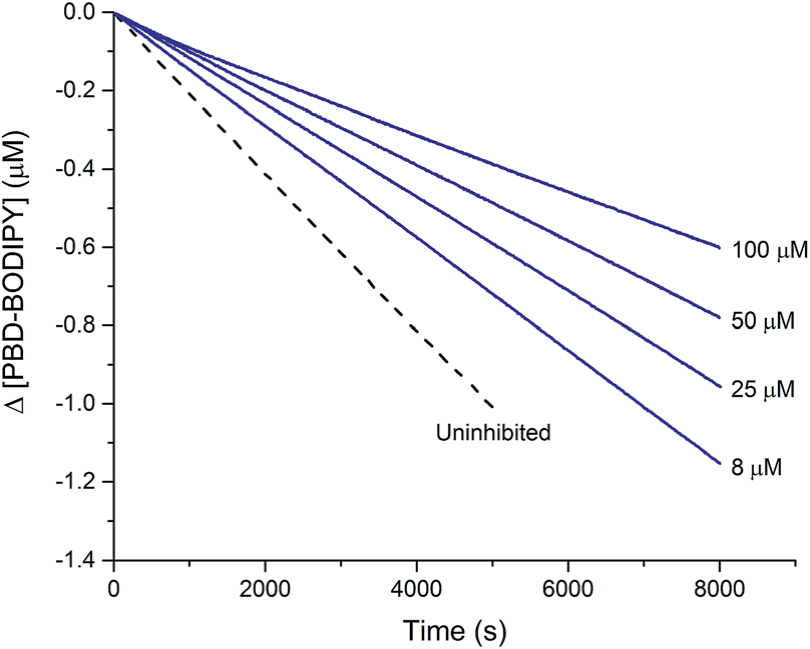
Thermally-initiated (AIBN, 6 mM) co-autoxidation of styrene (4.4 M) and PBD-BODIPY (10 μM) at 37 °C in chlorobenzene in the presence of various concentrations of *t*-BuS(O)SSS*t*-Bu.

This reactivity was subsequently confirmed in styrene autoxidations inhibited by *t*-BuS(O)SSS*t*-Bu, which yielded data that were indistinguishable from those wherein *t*-BuS(O)SS*t*-Bu was used as the inhibitor of (*cf.* Fig. 7), and characterized by a *k*_inh_ = (1.0 ± 0.3) × 10^4^ M^–1^ s^–1^. Although no stoichiometric information could be gleaned from the styrene autoxidations inhibited by *t*-BuS(O)SSS*t*-Bu (as was the case with *t*-BuS(O)SS*t*-Bu, *vide supra*), it is highly likely based on the foregoing that it also traps a single peroxyl radical by a mechanism analogous to that shown in Scheme 5. Similarly to the trisulfide, the tetrasulfide displayed no such RTA activity.

It seems reasonable to suggest that the reactivity of trisulfide-1-oxides and tetrasulfide-1-oxides extends to higher polysulfide-1-oxides, since the S–S bond strength in higher polysulfides is essentially independent of the number of sulfur atoms.^48^ The generality of this reactivity is in sharp contrast to that of the lower thiosulfinates and sulfoxides, whose activities require appropriate substitution to promote Cope-type elimination of a sulfenic acid.

## Conclusions

The RTA activity of trisulfides, tetrasulfides and their corresponding 1-oxides were determined by inhibited autoxidations. In each case, the polysulfides were inactive, while their 1-oxides were as reactive as hindered phenols – the most common type of primary antioxidant – with *k*_inh_ ∼ 1 to 2 × 10^4^ M^–1^ s^–1^. Kinetic isotope effects, steric effects and high level computations are consistent with a bimolecular homolytic substitution mechanism wherein the peroxyl radical is the nominal nucleophile, attacking the 
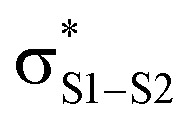
 of the polysulfide-1-oxide. The stoichiometry of the reaction of the trisulfide-1-oxides with peroxyl radicals was determined to be *n* ∼ 1, indicating that the peroxysulfinate ester initially formed rearranges to a sulfonate ester instead of cleaving to yield an alkoxyl radical that would propagate the chain. The other substitution product, the perthiyl radical, simply combines with another of itself to form a tetrasulfide. It is anticipated that higher polysulfide-1-oxides undergo the same rapid bimolecular homolytic substitution reactions with peroxyl radicals. This chemistry may contribute to the antioxidant activity of *Allium*-derived polysulfides, such as garlic's diallyltrisulfide, as well as that of sulfurized olefins that are added as secondary antioxidants to petroleum-derived products. Results of further investigations along these lines will be reported in due course.

## Experimental section

### I. General

All chemicals and solvents were purchased from commercial suppliers and used without further purification unless otherwise indicated. PBD-BODIPY and STY-BODIPY were synthesized following our previously reported procedure.^26^ Styrene and cumene were extracted three times with NaOH (1 M), washed with water, dried with MgSO_4_, filtered, distilled under reduced pressure and percolated through a silica column. They could be kept at –20 °C under nitrogen for up to 5 days. Immediately before use, the distilled material was percolated again through a silica/basic alumina (1 : 4) column. Chlorobenzene was dried over 3 Å molecular sieves before use. SCl_2_ was distilled in the presence of PCl_5_ and used within 2 hours. BnSSSBn, *t*-BuSSS*t*-Bu and *t*-BuSSSS*t*-Bu were prepared according to a previously reported procedure.^23^ Similarly, BnS(O)SBn,^16^ BnS(O)SSBn,^24^ *t*-BuS(O)SS*t*-Bu,^25^ BnSSH^32^ and *t*-BuS(O)SSS*t*-Bu^49^ were prepared according to previously reported procedures. UV-visible spectra were measured with a spectrophotometer equipped with a thermostatted 6 × 6 multi cell holder.

#### *d*_4_-BnSSSBn

Potassium carbonate (2.35 g, 17.0 mmol) was added portion-wise to a stirred solution of *d*_2_-BnSAc^13^ (2.35 g, 14.0 mmol) in methanol (40.0 mL). After 1 hour, it was extracted with ether. The organic layer was then washed with water and brine, dried with MgSO_4_, filtered and the solvent removed under reduced pressure to yield crude *d*_2_-BnSH as a pale yellow oil (1.66 g, 94%), which was used without purification. Freshly distilled SCl_2_ (411 mg, 4.00 mmol) was added to dry ether (20.0 mL) and cooled to –78 °C under argon. One equivalent of *d*_2_-BnSH (504 mg, 4.00 mmol) and triethylamine (404 mg, 4.00 mmol) in ether (10.0 mL) were added dropwise to the cooled SCl_2_ solution over a period of 30 minutes. The solution was stirred for another 30 minutes after the addition was completed. A second equivalent of *d*_2_-BnSH and triethylamine (404 mg, 4.00 mmol) in ether (10.0 mL) were then added dropwise to the cooled solution over a period of 30 minutes. The solution was stirred for another 30 minutes after the addition was completed. After completion of the reaction (as judged by TLC analysis), the reaction mixture was diluted with ether, washed with water, saturated aqueous NaHCO_3_ and water until the pH of the organic phase was *ca.* 7. The organic layer was dried with MgSO_4_, filtered and was removed under reduced pressure. The crude dark orange oil was recrystallized from ethanol to afford the final product as white crystals (767 mg, 68%). ^1^H NMR (400 MHz; *d*_6_-acetone): *δ* 7.38–7.28 (m, 10H). ^13^C NMR (100 MHz; acetone-d_6_): *δ* 137.67, 130.31, 129.41, 128.35. HRMS EI: *m*/*z* calc. for C_14_H_10_D_4_S_3_ 282.05087, found: 282.05220.

#### *d*_4_-BnS(O)SSBn

A solution of *m*-CPBA (155 mg, 0.90 mmol) in CH_2_Cl_2_ (2.00 mL) was added dropwise to a solution of *d*_4_-BnSSSBn (282 mg, 1.00 mmol) in CH_2_Cl_2_ (1.50 mL) at 0 °C. The solution was stirred at 0 °C for 20 minutes after which all the trisulfide was consumed (as judged by TLC). The reaction mixture was diluted with CH_2_Cl_2_ and washed with saturated aqueous NaHCO_3_, water and brine. The organic layer was dried over MgSO_4_ and removed under reduced pressure. The crude white solid was recrystallized from hexanes and ether to afford the final product as white crystals (229 mg, 77%) ^1^H NMR (400 MHz; CDCl_3_): *δ* 7.38–7.27 (m, 10H). ^13^C NMR (100 MHz; CDCl_3_): 136.60, 130.45, 129.96, 129.23, 128.94, 128.84, 127.90. HRMS ESI [M + Na]^+^: *m*/*z* calc. for C_14_H_10_D_4_OS_3_Na 321.03555, found: 321.03790.

### II. Inhibited co-autoxidations

The inhibited autoxidations were carried out following our recently published procedure.^26^ To a 3.5 mL quartz cuvette was added styrene or cumene (1.25 mL) along with PhCl, such that the final reaction volume is 2.50 mL. The cuvette was then pre-heated in a thermostatted sample holder of a UV-vis spectrophotometer and allowed to equilibrate to 37 °C for approximately 15 minutes. A small volume (12.5 μL) of a 2.00 mM solution of the BODIPY probe in 1,2,4-trichlorobenzene was added, followed by the appropriate amount of 0.300 M solution of AIBN in PhCl to achieve the required rate of initiation (typically 50 μL, where *R*_i_ = 2.2 × 10^–9^ M s^–1^). The solution was thoroughly mixed. The absorbance at either 591 nm (PBD-BODIPY) or 571 nm (STY-BODIPY) was monitored for 30–45 min to ensure that the reaction was proceeding at a constant rate, after which 10.0 μL of a solution of the test antioxidant was added. The solution was thoroughly mixed and the absorbance readings resumed. The resulting data was processed as previously reported.^26^ The rate of initiation (*R*_i_ = 1.1 × 10^–8^ M s^–1^) and 2*ek*_d_ = 2.8 × 10^–7^ M^–1^ s^–1^ for 40 mM AIBN in cumene, necessary to derive stoichiometric data, was determined using PMC as a standard, which has an established stoichiometry of 2.^40^

### III. Laser flash photolysis

Nanosecond transient absorption experiments were performed on an LFP-112 spectrometer (Luzchem, Canada). Excitation was performed using an EX10 (GAM Laser, USA) XeCl Excimer laser (308 nm, *ca.* 10 mJ per pulse, *ca.* 12 ns pulse width). The transient absorption spectra were recorded in a quartz cuvette (1 cm × 1 cm) equipped with a septum and samples were bubbled with nitrogen or oxygen for 10 minutes before measurement.

### IV. Calculations

Calculations were carried out using CBS-QB3 complete basis method^28^ as it is implemented in the Gaussian 09 suite of programs.^50^ CBS-QB3(+) calculations^39^ were performed for the perthiyl-O_2_ adduct, wherein the geometry of the adduct was optimized at the B3LYP/6-311+G(2d,d,p) level of theory and the resulting frequencies read into the CBS-QB3 calculation of Gaussian 09 without further modifications. Due to the large size of the structure, calculations for the transition states of the reactions of trisulfide-1-oxide and peroxyl radicals with *t*-butyl substituents were carried out with only the first two steps of the CBS-QB3 calculation (B3LYP/CBSB7).
