# Supplementary material for: Polysulfide-1-oxides react with peroxyl radicals as quickly as hindered phenolic antioxidants and do so by a surprising concerted homolytic substitution
Source: Chem Sci. 2016 Jun 23;7(10):6347–56. doi: 10.1039/c6sc01434h (PMC5450444; doi:10.1039/c6sc01434h)
Supplement: Supplementary file 1 [file SC-007-C6SC01434H-s001.pdf]

Electronic Supplementary Material (ESI) for Chemical Science.
This journal is © The Royal Society of Chemistry 2016

Polysulfide-1-Oxides React with Peroxyl Radicals as Quickly as Hindered Phenolic Antioxidants and do so by a Surprising Concerted Homolytic Substitution at Sulfur

Jean-Philippe R. Chauvin, Evan A. Haidasz, Markus Griesser and Derek A. Pratt*

Department of Chemistry and Biomolecular Sciences, University of Ottawa,
Ottawa, Ontario, CANADA K1N 6N5

*dpratt@uottawa.ca

Supporting Information

Table of Contents

Deuterium Kinetic Isotope Effects……………………….……………………………. S2

Laser Flash Photolysis of *t*-BuSSSS*t*-Bu…………………..…………………………... S2

Dependence of k_{inh} on the Rate of Initiation.. S3

Background Reaction of STY-BODIPY and PBD-BODIPY with Polysulfides............. S3

NMR Spectra……………………........……………...………………………..……... S4

Computational Data………………...……………………………………...……..…. S7

Deuterium Kinetic Isotope Effects

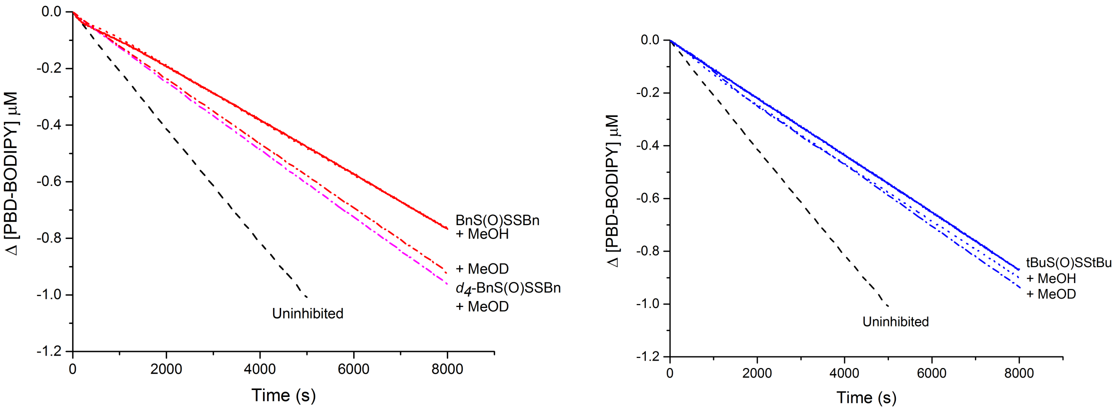

Figure S1. Thermally initiated (AIBN, 6 mM) co-autoxidations of styrene (4.35 M) and PBD-BODIPY (10 µM) at 37°C in chlorobenzene inhibited by either (d_4-) BnS(O)SSBn (left) or *t*-BuS(O)SS*t*-Bu (right) in the presence of 1% (v/v) MeOH or MeOD.

Laser Flash Photolysis of *t*-BuSSSS*t*-Bu

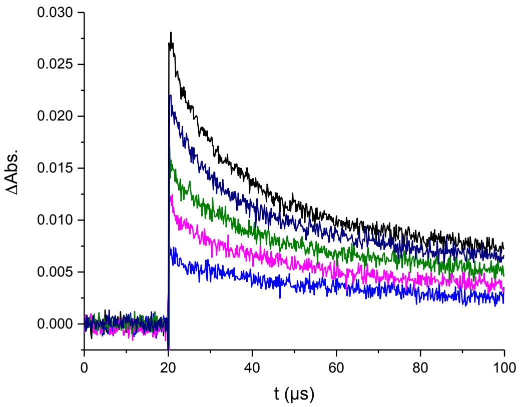

Figure S2. Transient absorption decays at 390 nm following laser flash photolysis at 308 nm of a 0.2 mM solution of di-*tert*-butyltetrasulfide in chlorobenzene at 22°C as a function of decreasing laser energies between 6 and 12 mJ/pulse.

Dependence of k_{inh} on the Rate of Initiation

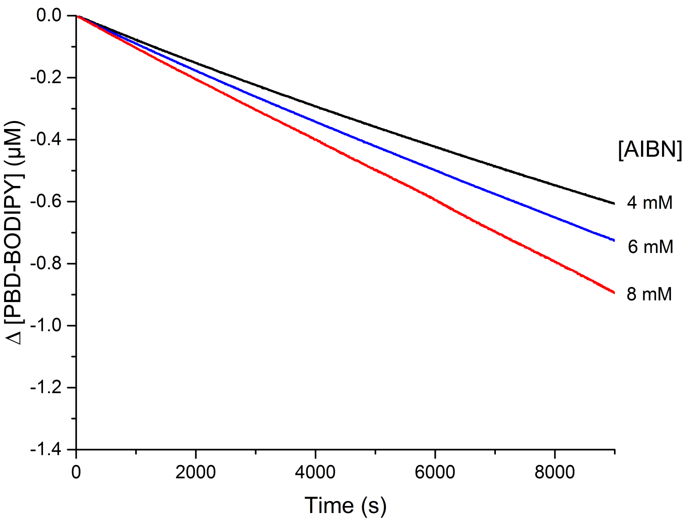

Figure S3. Thermally initiated (AIBN, 4, 6 or 8 mM) co-autoxidations of styrene (4.35 M) and PBD-BODIPY (10 μM) at 37°C in chlorobenzene inhibited by *t*-BuS(O)SS*t*-Bu (50 μM). The k_{inh} values obtained from these data are 1.4×10^4 $M^{-1}s^{-1}$, 1.3×10^4 $M^{-1}s^{-1}$ and 1.6×10^4 $M^{-1}s^{-1}$, for the AIBN concentrations of 4 mM, 6 mM and 8 mM, respectively.

Background Reaction of STY-BODIPY and PBD-BODIPY with Polysulfide

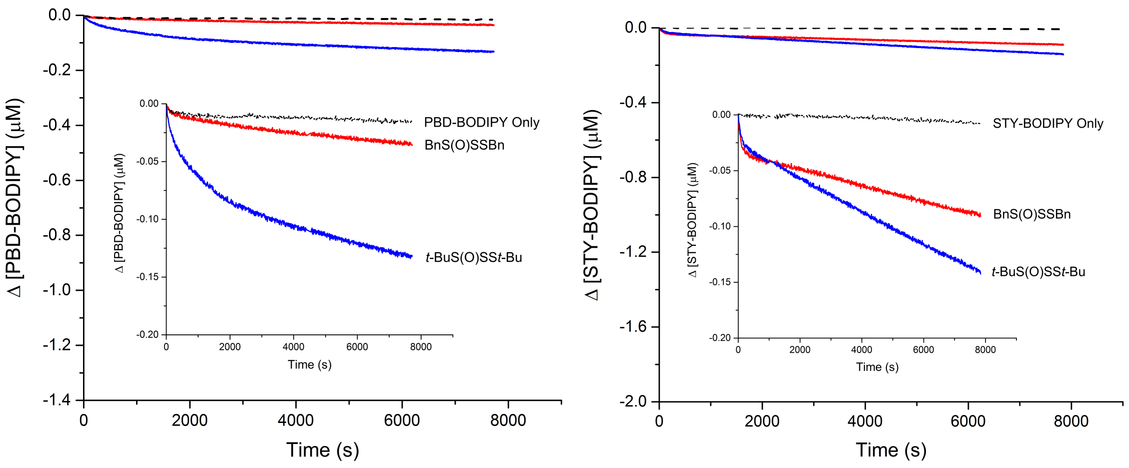

Figure S4. Decay of PBD-BODIPY (10 μM, left) and STY-BODIPY (10 μM, right) in PhCl containing cumene (3.6 M) at 37°C in the presence of the trisulfide-1-oxides (50 μM) and in the absence of initiator (compare to Figure 1C and 1D in the main text).

NMR Spectra

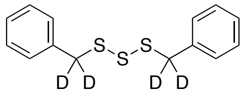

in d_6-acetone

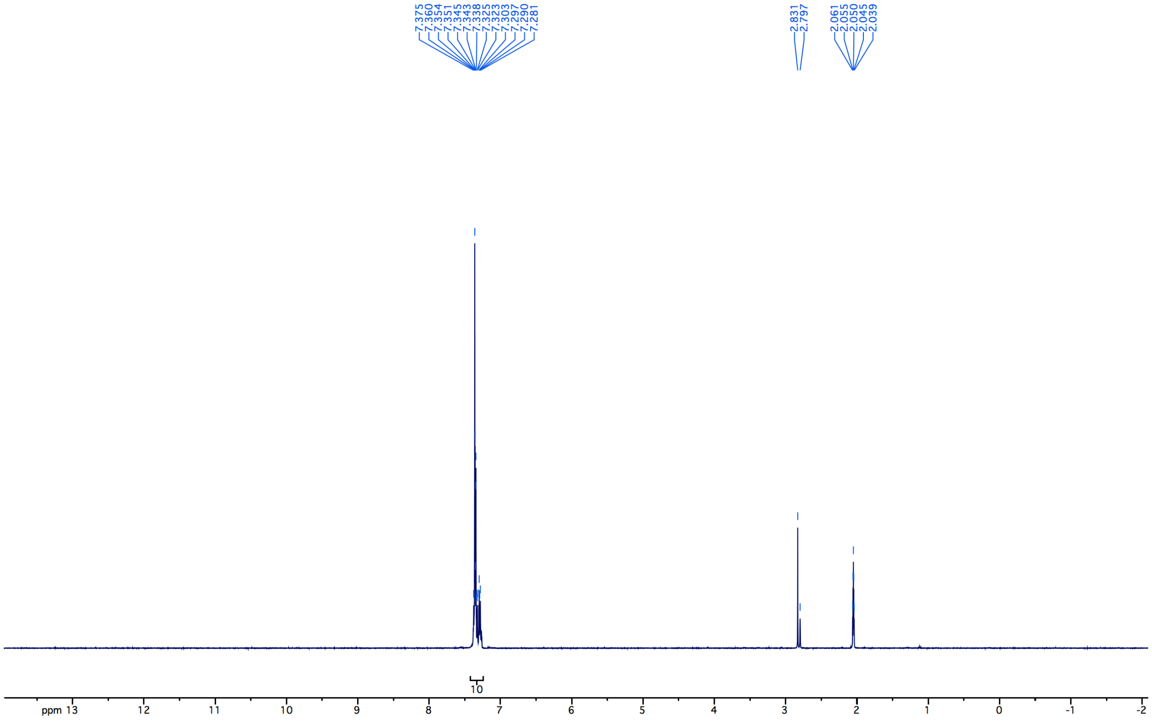

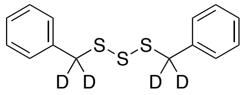

in d_6-acetone

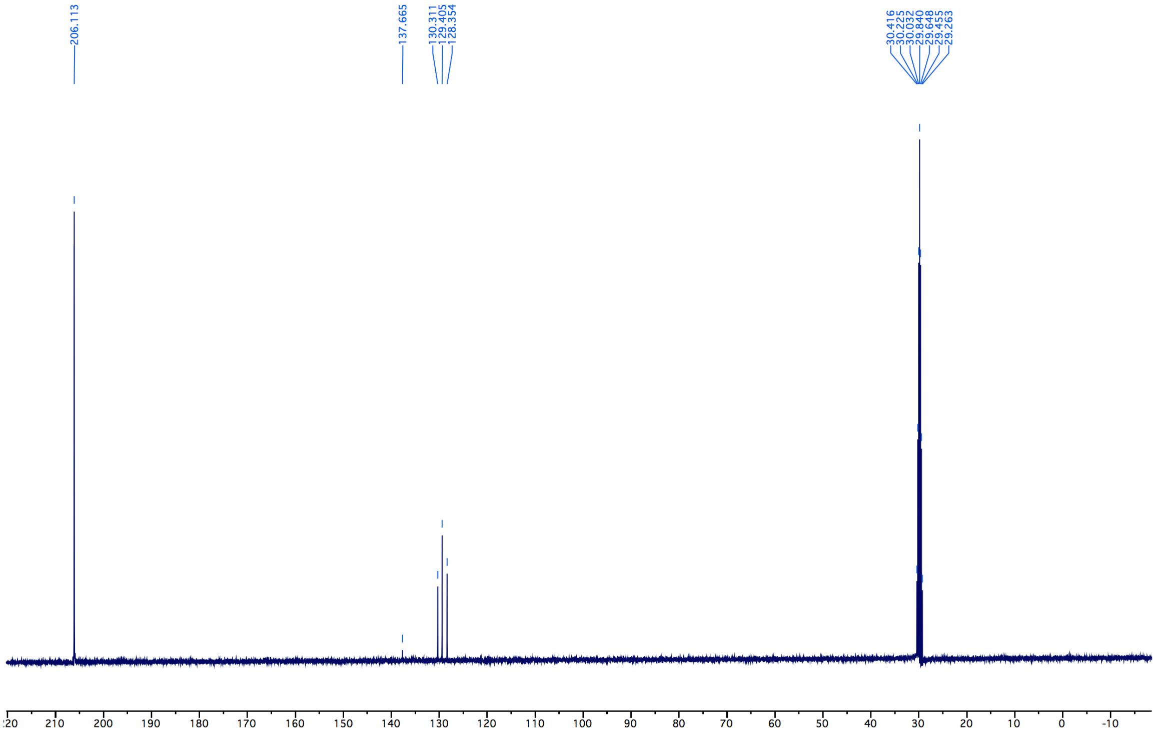

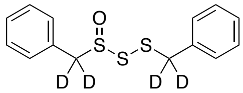
O
S
S
S
D D
D D

in $CDCl_3$

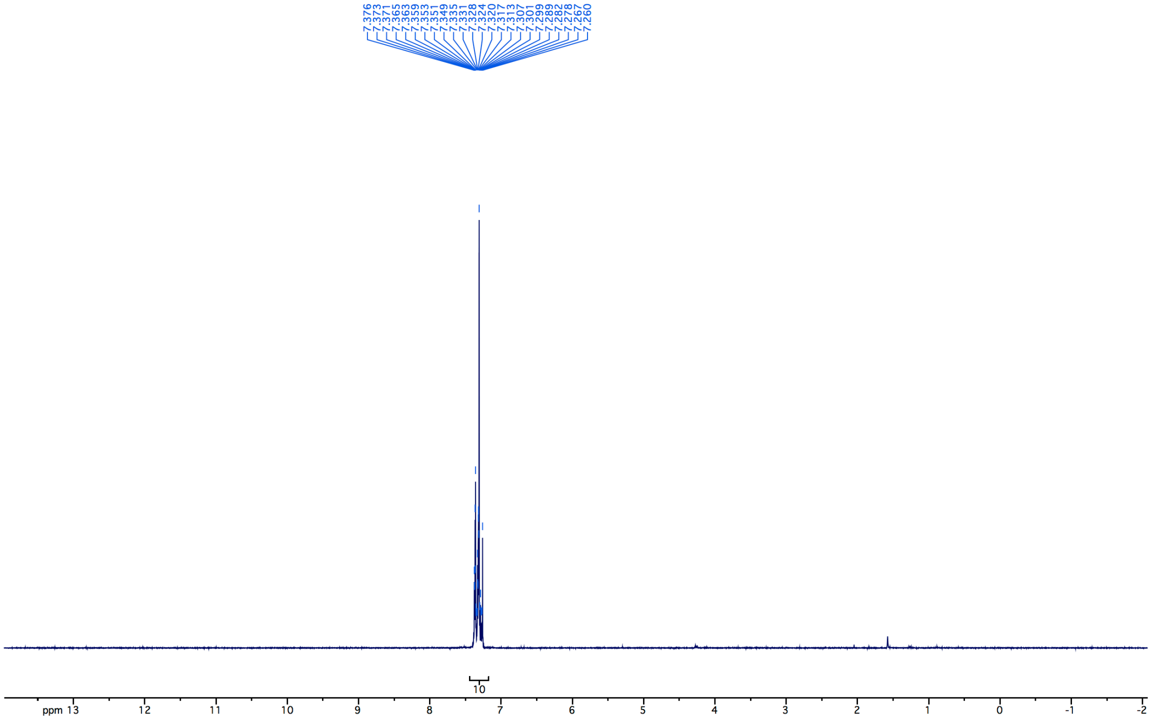
7.376
7.373
7.371
7.365
7.363
7.359
7.353
7.351
7.349
7.335
7.331
7.328
7.324
7.320
7.317
7.313
7.307
7.301
7.299
7.289
7.282
7.278
7.267
7.260
10
ppm 13
12
11
10
9
8
7
6
5
4
3
2
1
0
-1
-2

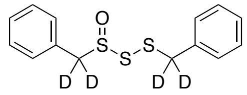

in $CDCl_3$

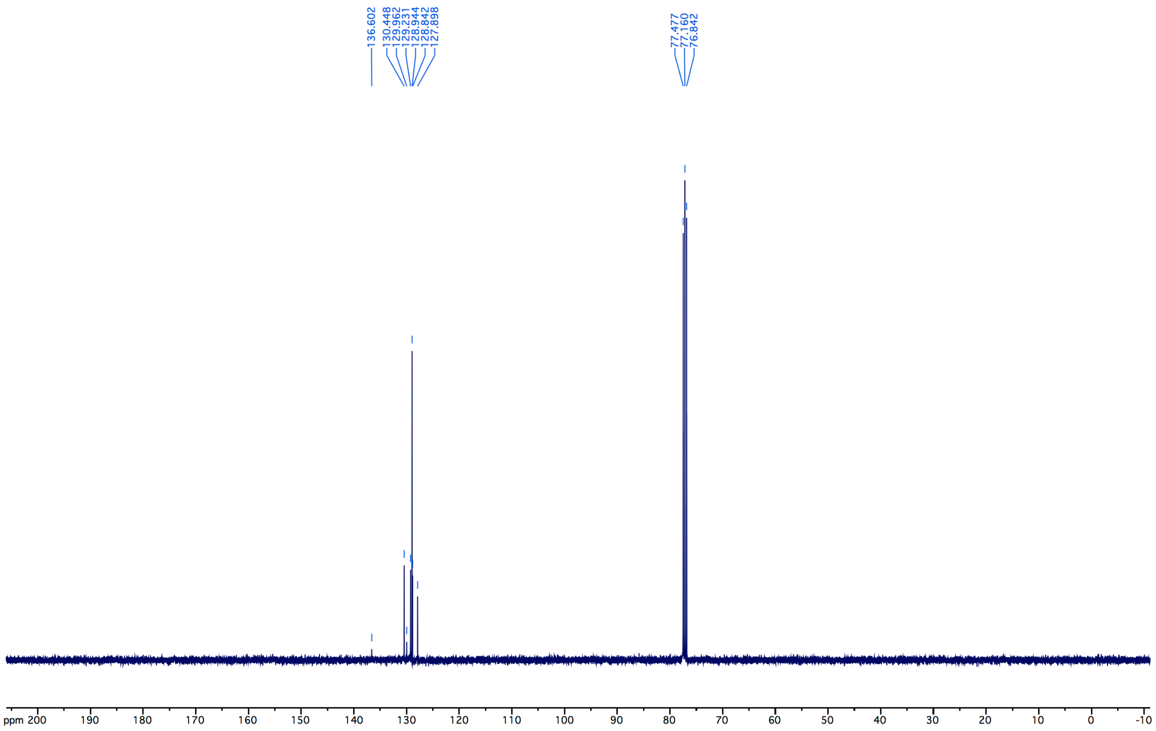
136.602
130.448
129.962
129.231
128.944
128.842
127.898
77.477
77.160
76.842
ppm 200
190
180
170
160
150
140
130
120
110
100
90
80
70
60
50
40
30
20
10
0
-10

Computational Data

Optimized Gaussian Structures and CBS-QB3 Energies

Dimethyltrisulfide-1-Oxide

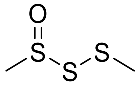

CBS-QB3 Enthalpy= -1348.008898 CBS-QB3 Free Energy= -1348.054027

```
0 1
 C                  -1.86937500   -0.52708900    1.24321100
 S                   0.29468400   -0.97164800   -0.87680800
 S                   1.61593100   -0.42494200    0.60996900
 C                   2.27647700    1.17506400    0.00589000
 S                  -1.55597200    0.19200300   -0.40134200
 O                  -1.19508900    1.61223500   -0.14834500
 H                  -2.72902000    0.00994300    1.64703300
 H                  -0.98983400   -0.36051600    1.86676200
 H                  -2.08787700   -1.58954400    1.13425000
 H                   1.47635300    1.90866400   -0.03763300
 H                   3.02762000    1.47644900    0.73988500
 H                   2.74657600    1.04266800   -0.96724100
```

Dimethyldisulfide-1-Oxide

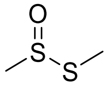

CBS-QB3 Enthalpy= -950.248208 CBS-QB3 Free Energy= -950.288074

```
0 1
 S                  -0.89343100    0.07738300   -0.52616100
 O                  -0.96896100    1.46807300    0.00400300
 S                   1.21622600   -0.53405400   -0.53621000
 C                  -1.48090200   -0.98515000    0.84298800
 H                  -1.32474600   -2.02970100    0.57349200
 H                  -0.94169500   -0.72663200    1.75374400
 H                  -2.54453400   -0.76945800    0.96172400
 C                   1.77243600    0.48348700    0.87191700
 H                   1.07323600    1.31998300    0.96976200
 H                   1.81626300   -0.09475100    1.79402800
 H                   2.75923800    0.87269200    0.62372000
```

Dimethyltetrasulfide-1-Oxide

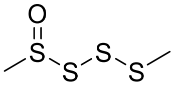

CBS-QB3 Enthalpy= -1745.767356 CBS-QB3 Free Energy= -1745.816046

0 1			
C	-2.27090000	0.10076200	1.37983500
S	-0.57574300	-1.31034900	-0.74386200
S	0.97603900	-1.09912000	0.64204000
S	-1.98353400	0.36687400	-0.40088800
O	-1.29840000	1.68421400	-0.50336800
H	-2.93301600	0.90795100	1.69635100
H	-1.31916400	0.16124200	1.90843300
H	-2.74366400	-0.87082100	1.52299100
S	2.45054300	0.08475900	-0.21622800
C	1.92991100	1.78324800	0.21300000
H	1.96477100	1.93332300	1.29141100
H	2.65763500	2.43900400	-0.27128300
H	0.92968300	1.97691700	-0.17496800

Dimethyltrisulfide

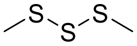

CBS-QB3 Enthalpy= -1272.886461 CBS-QB3 Free Energy= -1272.929472

0 1			
C	-2.45430000	0.74074600	-0.62072200
S	-0.00000700	-0.00013100	1.06378000
S	1.51221300	0.75707300	-0.16339000
C	2.45430600	-0.74059400	-0.62088300
S	-1.51221100	-0.75703100	-0.16356200
H	-3.30209100	0.39368200	-1.21600600
H	-1.83796400	1.40687700	-1.22353500
H	-2.81721900	1.25125900	0.27007800
H	1.83800900	-1.40655800	-1.22392100
H	3.30214200	-0.39338200	-1.21601700
H	2.81716200	-1.25135700	0.26980000

Dimethyl Disulfide

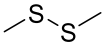

CBS-QB3 Enthalpy= -875.127093 CBS-QB3 Free Energy= -875.164992

0 1			
C	-1.87412100	0.79506000	0.38098900
S	0.91297000	-0.49323100	0.49584500
S	-0.91291800	-0.49300000	-0.49596500
H	-2.87315300	0.77318900	-0.06149300
H	-1.43917600	1.78311200	0.23367500
H	-1.94239200	0.56202000	1.44253100
C	1.87407000	0.79531900	-0.38080200
H	1.43898100	1.78318400	-0.23292800
H	2.87319800	0.77337100	0.06140600
H	1.94201700	0.56253800	-1.44239100

Dimethyltetrasulfide

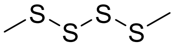

CBS-QB3 Enthalpy= -1670.643679 CBS-QB3 Free Energy= -1670.691739

0 1			
S	-0.69653500	0.79818100	-0.74394300
S	-2.08503000	0.45686300	0.76282200
C	-3.22970700	-0.75203800	0.00784300
S	0.69653400	-0.79813200	-0.74398900
H	-2.72074200	-1.69906700	-0.16785700
H	-4.02939900	-0.90098800	0.73713300
H	-3.64716500	-0.35939500	-0.91805200
S	2.08502500	-0.45690600	0.76279700
C	3.22971700	0.75202500	0.00788600
H	4.02940600	0.90093300	0.73718800
H	3.64717700	0.35942400	-0.91802600
H	2.72075900	1.69906600	-0.16777000

Dimethyltrisulfide-1-Oxide – S1 Transition State

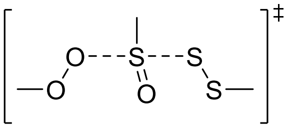

CBS-QB3 Enthalpy= -1537.956688 CBS-QB3 Free Energy= -1538.015224

```
0 2
 O                 -2.68617200    0.58813600   -0.04726400
 O                 -3.30143200   -0.37401600   -0.80001500
 C                 -3.52586700   -1.55054000   -0.00608700
 H                 -4.12990100   -1.29925900    0.86861000
 H                 -4.06737800   -2.23236800   -0.66273100
 H                 -2.57395900   -1.98575200    0.30286300
 S                  1.70137500    0.91069200   -0.89541700
 C                 -0.63489800    1.99632000    0.94210300
 H                 -0.58870000    2.80748400    0.21412900
 H                 -1.58625500    2.01203400    1.47294000
 H                  0.20767600    2.03634100    1.62925300
 S                  2.59458400   -0.87431600   -0.57471400
 C                  3.05333500   -0.85223800    1.19724500
 H                  3.59293200   -1.78478500    1.37831000
 H                  3.70201400   -0.00220000    1.40448600
 H                  2.14867600   -0.82210000    1.79978800
 S                 -0.54972800    0.41246400    0.04839900
 O                 -0.26242400   -0.64813100    1.03984100
```

Dimethyltrisulfide-1-Oxide – S2 Transition State

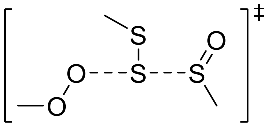

CBS-QB3 Enthalpy= -1537.949651 CBS-QB3 Free Energy= -1538.009738

```
0 2
 C                 -2.06951200   -1.76256000    1.13748500
 S                  0.30387200   -0.13387600   -0.46740400
 S                 -0.06157300    1.34687000    0.88324100
 C                 -0.59627400    2.75057500   -0.16324900
 H                 -3.07358700   -2.14061900    1.33763700
 H                 -1.81410300   -0.97336900    1.84566700
 H                 -1.33835300   -2.57166700    1.17214600
 H                 -1.52528600    2.49735900   -0.66887700
 H                 -0.75721900    3.58813200    0.51950000
 H                  0.19072400    2.99973600   -0.87287900
 O                  2.37276700    0.17522400   -0.85984000
 O                  3.17074600   -0.06188300    0.22774600
 C                  3.65027100   -1.40863400    0.18889200
 H                  4.15875200   -1.60128500   -0.75820600
 H                  4.34601700   -1.49183300    1.02495100
 H                  2.81646800   -2.10524400    0.31927300
 S                 -2.07152200   -1.05755200   -0.53997000
 O                 -2.99885800    0.11608800   -0.48188700
```

Dimethyldisulfide-1-Oxide – S1 Transition State

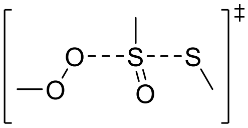

CBS-QB3 Enthalpy= -1140.198412 CBS-QB3 Free Energy= -1140.251298

0 2

O	-1.73949700	0.62823600	-0.58765300
O	-2.35867100	-0.59556200	-0.89019700
C	-3.15199700	-1.00295300	0.23200100
H	-3.90660800	-0.24515300	0.45812000
H	-3.63555100	-1.92370800	-0.09957500
H	-2.52692700	-1.19346000	1.10581300
S	2.34834900	0.06142200	-0.48012100
C	0.19718400	2.15479900	0.23363100
H	0.55824100	2.53973900	-0.72012100
H	-0.79864100	2.53414500	0.45544100
H	0.89689000	2.36683200	1.03897900
S	0.06273300	0.34698300	0.11482400
O	-0.08287800	-0.20982200	1.47060700
C	2.43272500	-1.67719400	0.05758300
H	3.47719900	-1.87777300	0.30331700
H	1.82747400	-1.81227000	0.95503000
H	2.11150000	-2.35355800	-0.73359200

Dimethyldisulfide-1-Oxide – S2 Transition State

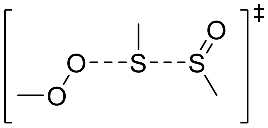

CBS-QB3 Enthalpy= -1140.191702 CBS-QB3 Free Energy= -1140.245209

0 2

C	1.84065600	1.86032600	0.14101400
S	-0.15792000	-0.47540000	-0.68771300
H	2.80653700	2.34041100	0.30724100
H	1.25099600	1.85488300	1.05710300
H	1.30052700	2.35842100	-0.66590200
O	-2.19745700	-0.40717100	-0.78082000
O	-2.80429000	-0.12315900	0.43382200
C	-3.14213600	1.26280700	0.47175500
H	-3.77137400	1.52395200	-0.38205700
H	-3.68866500	1.40103600	1.40627200
H	-2.23370200	1.87386000	0.47037400
S	2.16512500	0.13603600	-0.35636200
O	2.69315800	-0.56037300	0.86026900
C	-0.16566300	-1.69499100	0.66007300
H	-0.96883100	-1.42079100	1.34331400
H	-0.33613000	-2.68942500	0.25154700
H	0.79694000	-1.65574000	1.17410500

Dimethyltetrasulfide-1-Oxide – S1 Transition State

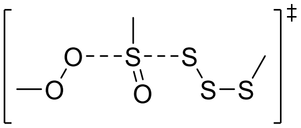

CBS-QB3 Enthalpy= -1935.714770 CBS-QB3 Free Energy= -1935.777827

0 2

O	3.20590000	-0.43340100	0.32370900
O	3.92470600	0.10547800	-0.71570200
C	4.09911300	1.51682000	-0.51151600
H	4.58606900	1.69739800	0.44919600
H	4.74128200	1.83801900	-1.33231700
H	3.13706000	2.03085400	-0.55224100
S	-0.86701400	-1.35916400	-1.00188300
C	1.06991900	-1.33894400	1.58846800
H	1.24692300	-2.37649700	1.30849800
H	1.84911000	-0.97255200	2.25615700
H	0.08226500	-1.20886100	2.02871800
S	-2.44227400	-0.92686800	0.21212400
S	1.14038100	-0.31229300	0.09261300
O	0.72433800	1.04667700	0.49775900
S	-3.32441300	0.87328500	-0.40373600
C	-2.45082600	2.11173300	0.62145000
H	-1.38137700	2.08200000	0.42087000
H	-2.86588800	3.07633400	0.31946500
H	-2.65110800	1.94625600	1.67919900

Dimethyltrisulfide – S1 Transition State

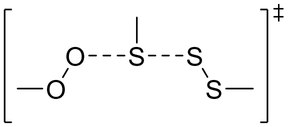

CBS-QB3 Enthalpy= -1462.829477 CBS-QB3 Free Energy= -1462.885362

0 2

S	-0.77749300	-0.97608400	-0.45590300
O	-2.57723800	-0.37794700	-0.44051900
O	-2.82165200	0.64505300	0.51068900
C	-2.83354900	1.89024200	-0.17703500
H	-3.57987900	1.88613000	-0.97555500
H	-3.09711100	2.62943300	0.58243900
H	-1.84360500	2.11336900	-0.58956100
S	1.57697700	-0.90190900	-0.77313100
C	-0.72919800	-1.39982900	1.31202100
H	-0.32909500	-0.57075600	1.89454900
H	-1.75579000	-1.60909800	1.60544500
H	-0.10761700	-2.28490400	1.43486300
S	2.42372100	0.29400600	0.65534100
C	2.61853800	1.90491600	-0.18973300
H	3.13937300	2.55662100	0.51562500
H	3.21579700	1.78786700	-1.09264200
H	1.64301600	2.32630400	-0.42895600

Dimethyltrisulfide – S2 Transition State

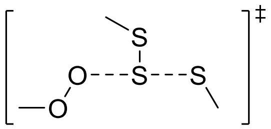

CBS-QB3 Enthalpy= -1462.817364 CBS-QB3 Free Energy= -1462.873724

```
0 2
 C                 -2.34928200   -1.52847600    1.07773600
 S                  0.07029100    0.05453100   -0.65368200
 S                 -0.30050900    1.50321200    0.77755500
 C                 -0.27785100    3.03161300   -0.22754900
 S                 -2.07874600   -1.16658900   -0.68267400
 H                 -3.31401100   -2.03680300    1.15007700
 H                 -2.38008400   -0.61221400    1.66784200
 H                 -1.57788300   -2.19943800    1.45703000
 H                 -1.07249200    3.01172500   -0.97131600
 H                 -0.45102000    3.84820900    0.47719600
 H                  0.69611100    3.15692300   -0.69686500
 O                  1.88997100    0.04197100   -0.71388800
 O                  2.50659000   -0.43402100    0.48435600
 C                  2.81874000   -1.80904300    0.29538800
 H                  3.46212800   -1.94853600   -0.57739000
 H                  3.35087100   -2.09791900    1.20429200
 H                  1.90766000   -2.40857400    0.19276100
```

Dimethyl Peroxysulfinate

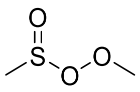

CBS-QB3 Enthalpy= -702.686388 CBS-QB3 Free Energy= -702.728013

```
0 1
 C                 -1.73686000    1.27559400    0.07874300
 H                 -2.72652600    1.16089300   -0.36325900
 H                 -1.80154400    1.27762200    1.16691200
 H                 -1.24208000    2.17225700   -0.29328000
 S                 -0.78871900   -0.20187100   -0.41565000
 O                 -1.49745800   -1.35191500    0.15303000
 O                  0.47830500    0.32717200    0.64178800
 O                  1.63224500   -0.50891500    0.35805000
 C                  2.57141500    0.30011900   -0.33758500
 H                  2.84945600    1.17584800    0.25539500
 H                  3.43901300   -0.35019100   -0.46902100
 H                  2.18912500    0.60849000   -1.31624800
```

Dimethyl Peroxydisulfane

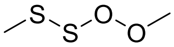

CBS-QB3 Enthalpy= -1025.306354 CBS-QB3 Free Energy= -1025.350627

0 1

C	-3.09042800	-0.05414500	-0.15597300
H	-3.53144900	-0.22895000	0.82979200
H	-2.94688600	1.01883900	-0.32665400
H	-3.74598000	-0.45787600	-0.93172900
S	0.06036800	0.90426800	0.24972400
S	1.60160900	0.00394900	-0.75958600
C	2.69491100	-0.59691100	0.58227400
O	-0.96517800	-0.32618800	0.75925500
O	-1.87845000	-0.77238400	-0.31475400
H	3.55448400	-1.05681200	0.08984900
H	2.17281400	-1.34361800	1.17760500
H	3.02750000	0.23185100	1.20512400

Dimethyl Peroxysulfide

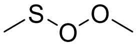

CBS-QB3 Enthalpy= -627.546951 CBS-QB3 Free Energy= -627.586276

0 1

C	2.30384400	-0.07696300	-0.35223800
H	2.81628400	-0.53841300	0.49726600
H	1.96125100	-0.84471200	-1.05586600
H	2.98511500	0.60470400	-0.86863000
S	-0.92936900	-0.69917500	-0.13195900
O	0.29388800	-0.12640300	0.83989700
O	1.23523500	0.74721100	0.07488500
C	-2.02891100	0.73983100	-0.17742100
H	-2.90854200	0.43052800	-0.74872300
H	-2.32746200	1.02092400	0.83210400
H	-1.53932500	1.57009000	-0.68510700

MeOOH

CBS-QB3 Enthalpy= -190.589541 CBS-QB3 Free Energy= -190.620494

0 1

C	1.12969900	-0.22351700	0.02691700
H	1.97288000	0.47152000	0.02385600
H	1.18943400	-0.87831400	-0.84803800
H	1.14425600	-0.82482500	0.94276100
O	-0.01622400	0.60681300	-0.03173700
O	-1.16383500	-0.28614900	-0.09022000
H	-1.64429200	0.00741100	0.69557200

MeOO•

CBS-QB3 Enthalpy= -189.954729 CBS-QB3 Free Energy= -189.985214

```
0 2
 C                 -1.00219900   -0.48042400    0.00000000
 H                 -1.96179900    0.03570300   -0.00000000
 H                 -0.88114200   -1.08868500    0.89703900
 H                 -0.88114200   -1.08868500   -0.89703900
 O                  0.00000000    0.56593800   -0.00000000
 O                  1.21716000    0.06208800    0.00000000
```

MeO•

CBS-QB3 Enthalpy= -114.870506 CBS-QB3 Free Energy= -114.897422

```
0 2
 C                  0.57337600   -0.00032600    0.01393900
 H                  0.87116800    0.01128100   -1.05452800
 H                  1.01227600   -0.91329600    0.44584000
 H                  1.01128800    0.90487200    0.46317600
 O                 -0.79187300   -0.00011300    0.00773500
```

MeSOH

CBS-QB3 Enthalpy= -513.274145 CBS-QB3 Free Energy= -513.306190

```
0 1
 C                  1.38405500    0.44605700    0.00142800
 S                 -0.08418500   -0.61206000    0.01045500
 H                  2.24006100   -0.23498200   -0.01873500
 H                  1.44429300    1.06783100    0.89628500
 H                  1.39883300    1.06499400   -0.89620600
 O                 -1.30913300    0.55452500   -0.11760100
 H                 -1.56749700    0.78257500    0.78361600
```

MeSO•

CBS-QB3 Enthalpy= -512.667753 CBS-QB3 Free Energy= -512.699932

```
0 2
 C                  1.40771700    0.30194500    0.00000900
 S                 -0.22733000   -0.50330800    0.00000600
 O                 -1.24810600    0.61086800    0.00000300
 H                  1.50591000    0.91897400    0.89437300
 H                  1.50475900    0.92070400   -0.89331900
 H                  2.16516400   -0.48536400   -0.00121900
```

MeSH

CBS-QB3 Enthalpy= -438.148250 CBS-QB3 Free Energy= -438.177063

```
0 1
 C                 1.16309500    0.02007000    0.00000400
 S                -0.66584100   -0.08682200    0.00000200
 H                 1.52436700   -1.00839100    0.00018400
 H                 1.52967900    0.52133800    0.89488600
 H                 1.52968800    0.52097100   -0.89508700
 H                -0.90884800    1.23481000   -0.00003000
```

MeS•

CBS-QB3 Enthalpy= -437.512005 CBS-QB3 Free Energy= -437.540433

```
0 2
 S                -0.69450100   -0.00001400    0.00199800
 C                 1.11164200   -0.00011700    0.00914200
 H                 1.51034600   -0.90289300    0.47265000
 H                 1.42167900    0.00658100   -1.04275600
 H                 1.51013500    0.89723800    0.48328600
```

MeSSH

CBS-QB3 Enthalpy= -835.902692 CBS-QB3 Free Energy= -835.936333

```
0 1
 C                 1.66413300    0.69033700   -0.00466900
 S                -1.37025000    0.24405500   -0.08762500
 S                 0.48562100   -0.70718500    0.01439900
 H                 2.65719500    0.23561700   -0.04731700
 H                 1.57838100    1.28780700    0.90237000
 H                 1.51163400    1.30981500   -0.88723800
 H                -1.57794100    0.43482100    1.23181000
```

MeSS•

CBS-QB3 Enthalpy= -835.292390 CBS-QB3 Free Energy= -835.325969

```
0 2
 S                -1.36215300    0.28656100   -0.00000200
 S                 0.37455500   -0.67757100    0.00000200
 C                 1.67072900    0.61597800    0.00000800
 H                 1.57226600    1.22938900    0.89456500
 H                 2.63348400    0.10062700   -0.00075600
 H                 1.57144000    1.23028000   -0.89385500
```

MeSSSH

CBS-QB3 Enthalpy= -1233.660462 CBS-QB3 Free Energy= -1233.699005

```
0 1
 S                  0.43701600   -0.79496700    0.49310600
 S                 -1.31092100   -0.38448800   -0.55019300
 C                 -2.05344900    0.98919700    0.39972700
 H                 -1.43703300    1.88402200    0.31932000
 H                 -3.02705500    1.17835500   -0.05845600
 H                 -2.18876000    0.70490000    1.44206100
 S                  1.91293300    0.57884700   -0.12377100
 H                  2.34910000   -0.09274500   -1.20755100
```

MeSSS•

CBS-QB3 Enthalpy= -1233.047112 CBS-QB3 Free Energy= -1233.086366

```
0 2
 S                  1.77651500    0.61454200   -0.15680200
 S                  0.59054600   -0.89961100    0.26426700
 S                 -1.43843100   -0.48026600   -0.27260300
 C                 -1.63137300    1.24844200    0.26440900
 H                 -0.85807400    1.86840100   -0.18856300
 H                 -2.60868600    1.56198100   -0.10651000
 H                 -1.60308200    1.32431600    1.35082300
```

MeSSOH

CBS-QB3 Enthalpy= -911.036351 CBS-QB3 Free Energy= -911.073227

```
0 1
 S                 -0.88085200   -0.57624400   -0.40341700
 O                  1.78032100    0.82037300   -0.38981700
 C                 -1.86999000    0.81223700    0.27068400
 H                 -2.86075300    0.73278100   -0.18232900
 H                 -1.95082000    0.72957700    1.35327900
 H                 -1.41392900    1.75968700   -0.01055400
 S                  0.94543700   -0.36324900    0.49635700
 H                  2.16951300    0.37344400   -1.15300100
```

MeSSO•

CBS-QB3 Enthalpy= -910.425022 CBS-QB3 Free Energy= -910.462592

```
0 2
 S                  1.13598400   -0.42095700    0.11566100
 S                 -0.98466500   -0.71572800   -0.09566700
 C                 -1.53832400    1.01132300    0.10792200
 O                  1.46693600    1.01121400   -0.17029300
 H                 -2.36609700    1.17127300   -0.58171500
 H                 -0.70732300    1.66722600   -0.15825700
 H                 -1.85322500    1.19081500    1.13487200
```

MeS(O)SH

CBS-QB3 Enthalpy= -911.025090 CBS-QB3 Free Energy= -911.061448

```
0 1
 S                  0.51627100   -0.25995700   -0.43690400
 S                 -1.58105400    0.09698100    0.13625600
 H                 -2.00056200   -0.98509300   -0.54256200
 O                  1.16503200   -1.23493500    0.46590700
 C                  1.04011900    1.39746800    0.14494000
 H                  0.53252000    2.17008100   -0.43231300
 H                  0.82647400    1.48240300    1.21038600
 H                  2.11713600    1.43488300   -0.02203600
```

MeS(S)OH

CBS-QB3 Enthalpy= -911.023380 CBS-QB3 Free Energy= -911.059386

```
0 1
 S                 -0.25207500   -0.06973200   -0.52159500
 C                 -1.22094600    1.26171800    0.24718700
 H                 -2.26696700    1.12263100   -0.02687500
 H                 -1.07367400    1.19285800    1.32370400
 H                 -0.83023500    2.19927600   -0.14427200
 O                 -1.13001000   -1.25157600    0.35727600
 H                 -0.71261600   -2.09824700    0.14331500
 S                  1.58015300    0.07134300    0.16927000
```

MeS(O)S•

CBS-QB3 Enthalpy= -910.402656 CBS-QB3 Free Energy= -910.438294

```
0 2
 S                  0.31816500   -0.21682800   -0.31679100
 S                 -1.58107900    0.12407400    0.09276800
 O                  1.04789800   -1.36438900    0.27093100
 C                  1.25630700    1.29009900    0.13013500
 H                  0.77587200    2.14067800   -0.34785100
 H                  1.23691500    1.37822300    1.21559200
 H                  2.27281700    1.13966900   -0.23163000
```

MeS(O)O•

CBS-QB3 Enthalpy= -587.802603 CBS-QB3 Free Energy= -587.836726

```
0 2
 C                 -1.58532700   -0.00214200    0.09436500
 O                  0.73980600   -1.28011100    0.20750300
 H                 -1.67586200   -0.00041900    1.18096400
 H                 -2.00855800   -0.90621800   -0.33792000
 H                 -2.01009900    0.90028400   -0.34006300
 S                  0.21278100    0.00012400   -0.27425800
 O                  0.73544200    1.28226300    0.20736700
```

H•

CBS-QB3 Enthalpy= -0.497457 CBS-QB3 Free Energy= -0.510472

Di-*tert*-Butyltrisulfide-1-Oxide

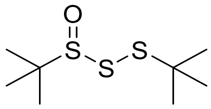

CBS-QB3 Enthalpy= -1583.378739 CBS-QB3 Free Energy= -1583.441926

```
0 1
 C                 -2.81121400   -0.04046300    0.18914200
 C                 -2.43717900   -0.18393800    1.66137300
 H                 -3.34598600   -0.29495100    2.26153400
 H                 -1.80753300   -1.05819000    1.83266200
 H                 -1.90266000    0.70375900    2.00588400
 C                 -3.32398400   -1.33807700   -0.43674800
 H                 -4.27893000   -1.61045000    0.02348000
 H                 -3.49900700   -1.22733200   -1.51167800
 H                 -2.62965300   -2.16555500   -0.27894600
 C                 -3.81048100    1.10966100   -0.01748100
 H                 -3.41519300    2.04818200    0.37536100
 H                 -4.05335700    1.25108400   -1.07456900
 H                 -4.74016700    0.87919000    0.51193000
 S                  0.11193400   -1.28344300   -0.46125800
 S                  1.39353700   -0.51134500    0.91854900
 C                  2.87521900    0.18483000   -0.02674300
 C                  2.43725100    1.23396500   -1.04767800
 H                  3.32510600    1.68870400   -1.50073000
 H                  1.85263600    0.77961800   -1.85066700
 H                  1.83122900    2.01408400   -0.58716200
 C                  3.70881600    0.82249800    1.09569100
 H                  3.97756800    0.09265700    1.86444500
 H                  4.63720400    1.21924500    0.67340400
 H                  3.17341300    1.64719800    1.57169500
 C                  3.63843500   -0.96064800   -0.69633500
 H                  4.51972400   -0.56463900   -1.21303600
 H                  3.97209800   -1.69927200    0.03584200
 H                  3.01538800   -1.46734800   -1.43670500
 S                 -1.29803500    0.51758100   -0.80940100
 O                 -0.70150500    1.65404500   -0.04953700
```

*ᵗ*BuSO•

CBS-QB3 Enthalpy= -630.352266 CBS-QB3 Free Energy= -630.393973

0 2

C	0.64511300	0.06007300	-0.00006000
C	0.78752600	0.91618000	-1.26378000
H	1.76058400	1.41802000	-1.26734400
H	0.71480200	0.30833500	-2.16938000
H	0.00668500	1.67856300	-1.29866200
C	1.60392600	-1.13493700	-0.00083400
H	2.63888200	-0.77998200	0.00029500
H	1.46697000	-1.76193200	0.88478200
H	1.46801300	-1.75995000	-0.88796800
C	0.78790800	0.91467200	1.26469400
H	0.00740800	1.67739100	1.30061500
H	0.71510800	0.30582600	2.16961200
H	1.76112000	1.41626300	1.26883600
S	-1.07247400	-0.68122200	-0.00007000
O	-2.04085300	0.48263600	0.00002700

^{t}BuSS•

CBS-QB3 Enthalpy= -952.978930 CBS-QB3 Free Energy= -953.021651

0 2

S	2.16160400	0.27291800	-0.00001300
S	0.59096700	-0.93910700	0.00010400
C	-0.98108900	0.11938000	0.00001000
C	-1.00833800	0.97944600	-1.26455000
H	-1.92455100	1.57894900	-1.27918300
H	-0.15587600	1.66076400	-1.29332500
H	-0.98889300	0.36405400	-2.16711600
C	-2.11308200	-0.91666000	-0.00071400
H	-2.07622800	-1.55557800	0.88547400
H	-3.07763900	-0.39980100	-0.00067100
H	-2.07589900	-1.55476300	-0.88748300
C	-1.00912100	0.97862100	1.26511100
H	-1.92541000	1.57800800	1.27964600
H	-0.99009000	0.36262600	2.16727600
H	-0.15676500	1.66003700	1.29479200

Diallyl Thiosulfinate (Allicin)

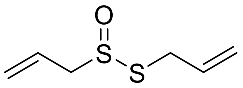

CBS-QB3 Enthalpy= -1104.729282 CBS-QB3 Free Energy= -1104.781288

```
0 1
 C                 -3.39551200    1.62682400    0.16608100
 H                 -4.15696400    1.02248600   -0.31742300
 H                 -3.74284300    2.42360900    0.81329000
 C                 -2.09635900    1.41030800   -0.03143200
 H                 -1.36336200    2.03312700    0.47164100
 C                 -1.55492600    0.31960700   -0.89136300
 H                 -0.69203200    0.61478100   -1.49056700
 H                 -2.31597100   -0.11071900   -1.54764800
 S                 -0.99069900   -1.16545700    0.09740900
 O                 -0.37058700   -2.07138600   -0.91162800
 H                  1.60080100   -1.13956800   -0.72212600
 S                  0.64014300   -0.25363300    1.26281200
 C                  1.99909200   -0.44214000    0.02655700
 H                  2.81303400   -0.94523800    0.55024300
 C                  2.44539200    0.85287800   -0.57314400
 H                  1.70989100    1.38393200   -1.17272800
 C                  3.66175300    1.36894100   -0.42038500
 H                  4.42036400    0.87115600    0.17591300
 H                  3.94403200    2.30446800   -0.88898600
```

Diallyl Thiosulfinate (Allicin) Cope Transition State

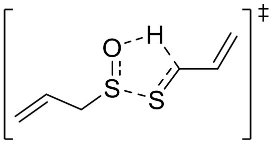

CBS-QB3 Enthalpy= -1104.699642 CBS-QB3 Free Energy= -1104.750167

```
0 1
 C                  3.94145500   -0.79242500   -0.36843800
 H                  4.41261800    0.04399800   -0.87555600
 H                  4.60004000   -1.50711000    0.11098500
 C                  2.61922100   -0.94381200   -0.34470700
 H                  2.17917000   -1.79098700    0.17322700
 C                  1.65784200    0.01391400   -0.97365400
 H                  0.82617900   -0.48568900   -1.47226600
 H                  2.14939600    0.69682300   -1.66945300
 S                  0.89442500    1.07114900    0.34001700
 O                 -0.31909100    1.75469600   -0.36908400
 H                 -1.25717500    1.04995400   -0.08970800
 S                 -0.65473000   -0.62695400    1.35703600
 C                 -1.98448100    0.01340500    0.49706900
 H                 -2.68208100    0.60333700    1.09235400
 C                 -2.57071500   -0.69474800   -0.65671200
 H                 -1.91789200   -1.40688100   -1.15622100
 C                 -3.81427700   -0.50956300   -1.10695400
 H                 -4.49674900    0.18619200   -0.62904000
 H                 -4.19015900   -1.05493900   -1.96410200
```

Diallyl Trisulfide-1-Oxide

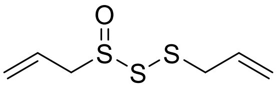

CBS-QB3 Enthalpy= -1502.490690 CBS-QB3 Free Energy= -1502.547798

```
0 1
 C                  4.44280600   -0.79098300   -0.05594100
 H                  4.97668900    0.11492400   -0.32649200
 H                  5.02916200   -1.59236900    0.37799200
 C                  3.13270500   -0.91955400   -0.25629600
 H                  2.63338600   -1.84060000    0.02851900
 C                  2.27009900    0.14855500   -0.83895600
 H                  1.51207800   -0.21986800   -1.53466600
 H                  2.84890400    0.93782600   -1.32460500
 O                  0.51814500    2.08537600   -0.31516200
 H                 -1.89644100    1.25577000   -0.56009500
 S                 -1.10312100   -1.01142800   -0.59714500
 C                 -2.41820300    0.30658500   -0.67431500
 H                 -2.78820600    0.23205300   -1.70093800
 C                 -3.51471300    0.11238900    0.31921900
 H                 -3.22853100    0.19906600    1.36409900
 C                 -4.77951700   -0.14478600   -0.00225800
 H                 -5.09947200   -0.23570000   -1.03592800
 H                 -5.54258400   -0.26930500    0.75726200
 S                  1.31626100    1.09191900    0.45269500
 S                 -0.04996500   -0.55161800    1.11491400
```

Diallyl Trisulfide-1-Oxide Cope-like Transition State

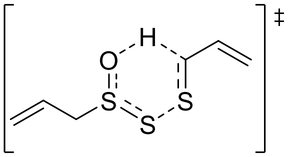

CBS-QB3 Enthalpy= -1502.425059 CBS-QB3 Free Energy= -1502.479262

```
0 1
 C                 -4.25903800   -0.52527900   -0.72357700
 H                 -4.38803700   -1.59643400   -0.60276600
 H                 -5.16136300    0.07104600   -0.78870900
 C                 -3.05339400    0.03358300   -0.80239200
 H                 -2.95745800    1.10785700   -0.92412000
 C                 -1.77557500   -0.73415300   -0.72501300
 H                 -1.02293100   -0.38289400   -1.43264600
 H                 -1.91949000   -1.81226500   -0.82315800
 O                  0.28790800   -1.43909200    0.86972600
 H                  1.08003400   -0.89319300    0.21186800
 S                  1.08086800    1.31609200   -0.83219200
 C                  1.96415100   -0.14511300   -0.69809800
 H                  1.75917900   -0.85395500   -1.50212600
 C                  3.30183400   -0.17865700   -0.10044200
 H                  3.55118900    0.66921800    0.53373900
 C                  4.19333500   -1.16518800   -0.25389500
 H                  3.98788900   -2.03249400   -0.87392700
 H                  5.16023400   -1.12868600    0.23254200
 S                 -1.01987900   -0.56276000    0.96699900
 S                 -0.34976300    1.41250300    1.04219300
```

1-*tert*-Butyl Methyltrisulfide-1-Oxide (B3LYP/CBSB7)

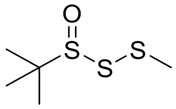

Sum of electronic and thermal Enthalpies= -1467.525025
Sum of electronic and thermal Free Energies= -1467.579067

0 1

C	-1.93655700	-0.10581100	0.23219200
C	-1.45198800	-0.27539100	1.66863300
H	-2.30912900	-0.46093400	2.32390100
H	-0.76354000	-1.11652600	1.76329500
H	-0.94624600	0.62980800	2.01033300
C	-2.42316400	-1.40998800	-0.40097000
H	-3.32422800	-1.75095600	0.11819300
H	-2.68436100	-1.27487800	-1.45536100
H	-1.67419500	-2.20060400	-0.32568500
C	-3.00670100	0.99374400	0.13417700
H	-2.63377800	1.94075900	0.52830800
H	-3.33238500	1.15167200	-0.89805400
H	-3.88228000	0.69741500	0.72008000
S	1.02612200	-1.10544600	-0.69458700
S	2.22635600	-0.36038800	0.78431100
S	-0.53556100	0.56068800	-0.85661700
O	0.05086900	1.71852700	-0.12016200
C	3.32488900	0.80313200	-0.10263100
H	2.74182900	1.64690900	-0.46357200
H	4.05275800	1.14304300	0.63817900
H	3.83906200	0.29430400	-0.91643900

1-*tert*-Butyl Methyltrisulfide-1-Oxide + MeOO• Transition State (B3LYP/CBSB7)

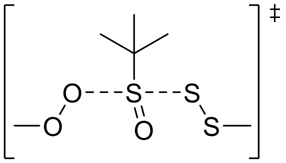

Sum of electronic and thermal Enthalpies= -1657.739036
Sum of electronic and thermal Free Energies= -1657.807161

```
0 2
 O                  2.50718100   -0.68216300    0.02874300
 O                  2.62619300   -1.91312800    0.62703700
 C                  2.50963500   -2.94373800   -0.36044300
 H                  3.24141400   -2.78757900   -1.15607300
 H                  2.71420900   -3.87282300    0.17344600
 H                  1.49889700   -2.96029600   -0.77743400
 S                 -1.67400400   -0.01966700    1.31644500
 C                  0.91703400    1.77800300   -0.01691900
 S                 -2.82858800   -1.20961000    0.15779400
 C                 -3.47820300   -0.10582200   -1.14983500
 H                 -4.16949400   -0.71362200   -1.73832500
 H                 -4.01086900    0.73238600   -0.70295700
 H                 -2.65688700    0.23287700   -1.77690500
 S                  0.42069500   -0.06029500   -0.10111700
 O                 -0.07777400   -0.30308300   -1.47432500
 C                  1.32506100    2.04372300    1.43324600
 H                  2.13657400    1.38140000    1.73963400
 H                  1.67425900    3.07686000    1.52614600
 H                  0.48672400    1.91756300    2.12336900
 C                  2.08785400    1.96575100   -0.99365700
 H                  1.81199300    1.63568900   -1.99721500
 H                  2.32835800    3.03267200   -1.04122500
 H                  2.97703500    1.42306800   -0.67783600
 C                 -0.27505200    2.62455900   -0.46969700
 H                 -1.11434300    2.56342500    0.22261700
 H                  0.04389000    3.66967100   -0.53476300
 H                 -0.61418000    2.31400500   -1.46025400
```

1-*tert*-Butyl Methyltrisulfide-1-Oxide + *t*BuOO• Transition State (B3LYP/CBSB7)

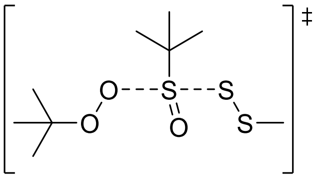

Sum of electronic and thermal Enthalpies= -1775.638303
Sum of electronic and thermal Free Energies= -1775.715440

```
0 2
 O                 -1.59352700    0.36075200    1.04727500
 O                 -2.53315200    0.37055500    0.03505600
 C                 -3.23617700   -0.92178700   -0.07416500
 S                  2.01310500   -0.67195900   -1.28353600
 C                  0.51886200    2.17829100   -0.19349500
 S                  2.75242700   -2.15409300   -0.11135800
 C                  4.04850800   -1.34818500    0.90004300
 H                  4.51145900   -2.14976400    1.48056500
 H                  4.79218000   -0.88329400    0.25431500
 H                  3.59198100   -0.62197100    1.56823600
 S                  0.41668700    0.35012700    0.35719400
 O                  1.11314100    0.25644400    1.65507000
 C                 -0.09370000    2.23750000   -1.59524100
 H                 -1.12175400    1.86998100   -1.59444000
 H                 -0.10709300    3.27727700   -1.93738300
 H                  0.48563700    1.66161700   -2.32180800
 C                 -0.28354700    3.00210500    0.82177200
 H                  0.07461300    2.82257300    1.83754800
 H                 -0.14022200    4.06301200    0.59217700
 H                 -1.34622600    2.77278800    0.78646400
 C                  1.99025100    2.60192800   -0.16270900
 H                  2.58764900    2.08467500   -0.91300200
 H                  2.04882100    3.67785400   -0.35618600
 H                  2.42466000    2.41045000    0.82053000
 C                 -3.94573800   -1.20506700    1.24958900
 H                 -4.50102600   -2.14464400    1.18783900
 H                 -3.21849900   -1.28211700    2.05856500
 H                 -4.64713500   -0.40180400    1.48699500
 C                 -2.24209800   -2.02252800   -0.43912200
 H                 -2.77387900   -2.96470600   -0.59567300
 H                 -1.70535000   -1.77505500   -1.35771600
 H                 -1.51551900   -2.17118900    0.36047000
 C                 -4.22634600   -0.65088300   -1.20550600
 H                 -3.70154800   -0.42707700   -2.13692900
 H                 -4.85550300   -1.52927800   -1.36666300
 H                 -4.87054200    0.19523200   -0.95690000
```

MeOO• (B3LYP/CBSB7)

Sum of electronic and thermal Enthalpies= -190.224259
Sum of electronic and thermal Free Energies= -190.254713

```
0 2
 C                 -1.00219900   -0.48042400    0.00000000
 H                 -1.96179900    0.03570300   -0.00000000
 H                 -0.88114200   -1.08868500    0.89703900
 H                 -0.88114200   -1.08868500   -0.89703900
 O                  0.00000000    0.56593800   -0.00000000
 O                  1.21716000    0.06208800    0.00000000
```

^{t}BuOO• (B3LYP/CBSB7)

Sum of electronic and thermal Enthalpies= -308.126174
Sum of electronic and thermal Free Energies= -308.166037

```
0 2
 O                  0.00000000    0.00000000    0.00000000
 C                  0.00000000    0.00000000    2.35427430
 O                  0.76990259    0.00000000    1.06396020
 C                  1.10883221    0.00015895    3.39913797
 H                  1.73849999    0.88649873    3.29558195
 H                  0.67484561    0.00032047    4.40175522
 H                  1.73853399   -0.88619650    3.29587481
 C                 -0.84501609    1.26886668    2.38991826
 H                 -1.53986348    1.28157290    1.54940564
 H                 -1.41835047    1.30961678    3.31934841
 H                 -0.21124546    2.15692960    2.33488271
 C                 -0.84463185   -1.26915647    2.39002096
 H                 -0.21054468   -2.15697958    2.33467659
 H                 -1.41768707   -1.31024625    3.31960830
 H                 -1.53971250   -1.28203738    1.54970147
```

^{t}BuSS• (B3LYP/6-311+G(2d,d,p) geometry)

CBS-QB3 Enthalpy= -952.979010 CBS-QB3 Free Energy= -953.021771

```
0 2
 S                  0.59124600   -0.93895300    0.00000700
 S                  2.16183100    0.27303800   -0.00000100
 C                 -0.98123900    0.11912900   -0.00000100
 C                 -1.00919500    0.97909200   -1.26472200
 H                 -1.92563800    1.57831200   -1.27895000
 H                 -0.15709300    1.66088100   -1.29410100
 H                 -0.99018900    0.36369100   -2.16729700
 C                 -1.00921000    0.97910500    1.26471400
 H                 -1.92564200    1.57833900    1.27891200
 H                 -0.99023900    0.36370900    2.16729400
 H                 -0.15709600    1.66087900    1.29411600
 C                 -2.11285300   -0.91718300   -0.00000300
 H                 -3.07759300   -0.40072400   -0.00001600
 H                 -2.07536900   -1.55566000   -0.88644700
 H                 -2.07538000   -1.55564200    0.88645400
```

O_2 (B3LYP/6-311+G(2d,d,p) geometry)

CBS-QB3 Enthalpy= -150.161267 CBS-QB3 Free Energy= -150.184540

```
0 3
 O                  0.00000000    0.00000000    0.60277200
 O                  0.00000000    0.00000000   -0.60277200
```

^{t}BuSSOO• (B3LYP/6-311+G(2d,d,p) geometry)

CBS-QB3 Enthalpy= -1103.134599 CBS-QB3 Free Energy= -1103.185777

```
0 2
 S                 -0.34328800   -0.91712100   -0.69587500
 S                  1.31271000   -0.88123800    0.41411600
 C                 -1.58579500    0.28508500    0.08417600
 O                  2.33832000    0.58705300   -0.44833000
 O                  3.46742600    0.79828500    0.07523000
 C                 -1.02414100    1.70694500    0.03763200
 H                 -1.76419100    2.40324800    0.44653000
 H                 -0.11481000    1.79422700    0.63539400
 H                 -0.79307500    2.01378300   -0.98454800
 C                 -2.81590300    0.14004900   -0.82353400
 H                 -3.20388400   -0.88199500   -0.82009500
 H                 -3.60926000    0.80031200   -0.46026400
 H                 -2.59225700    0.42227100   -1.85559400
 C                 -1.89674800   -0.15042300    1.51657200
 H                 -2.63834600    0.52825300    1.95100100
 H                 -2.30224100   -1.16429300    1.54810900
 H                 -1.00313600   -0.11470200    2.14332700
```

^{t}BuSSOO• Transition State (B3LYP/6-311+G(2d,d,p) geometry)

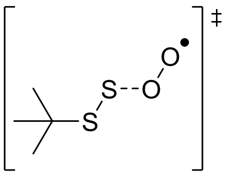

CBS-QB3 Enthalpy= -1103.133138 CBS-QB3 Free Energy= -1103.183605

```
0 2
 S                 -0.35950800   -0.88809600   -0.73112100
 S                  1.27515000   -0.94718700    0.40030700
 C                 -1.59375100    0.29501800    0.08973600
 O                  2.40692700    0.62330700   -0.42649400
 O                  3.52551800    0.80408300    0.09162200
 C                 -0.99962200    1.70362100    0.13619400
 H                 -1.73045700    2.39180100    0.57430800
 H                 -0.09863000    1.73308100    0.75198300
 H                 -0.74494700    2.06481000   -0.86243600
 C                 -2.80820400    0.23044500   -0.84753300
 H                 -3.21807000   -0.78108300   -0.91040400
 H                 -3.59448600    0.88669400   -0.46221100
 H                 -2.55761400    0.56574200   -1.85730000
 C                 -1.94173000   -0.21484600    1.48884800
 H                 -2.67700900    0.45424700    1.94818900
 H                 -2.36939700   -1.21953600    1.45445800
 H                 -1.05938700   -0.23576100    2.13195700
```
